# Novel Candidate Genes Associated with Hippocampal Oscillations

**DOI:** 10.1371/journal.pone.0026586

**Published:** 2011-10-31

**Authors:** Rick Jansen, Jaap Timmerman, Maarten Loos, Sabine Spijker, Arjen van Ooyen, Arjen B. Brussaard, Huibert D. Mansvelder, The Neuro-Bsik Mouse Phenomics Consortium, August B. Smit, Mathisca de Gunst, Klaus Linkenkaer-Hansen

**Affiliations:** 1 Department of Mathematics, VU University Amsterdam, Amsterdam, The Netherlands; 2 Department of Integrative Neurophysiology, Center for Neurogenomics and Cognitive Research, Neuroscience Campus Amsterdam, VU University Amsterdam, Amsterdam, The Netherlands; 3 Department of Molecular and Cellular Neurobiology, Center for Neurogenomics and Cognitive Research, Neuroscience Campus Amsterdam, VU University Amsterdam, Amsterdam, The Netherlands; Indiana University, United States of America

## Abstract

The hippocampus is critical for a wide range of emotional and cognitive behaviors. Here, we performed the first genome-wide search for genes influencing hippocampal oscillations. We measured local field potentials (LFPs) using 64-channel multi-electrode arrays in acute hippocampal slices of 29 BXD recombinant inbred mouse strains. Spontaneous activity and carbachol-induced fast network oscillations were analyzed with spectral and cross-correlation methods and the resulting traits were used for mapping quantitative trait loci (QTLs), i.e., regions on the genome that may influence hippocampal function. Using genome-wide hippocampal gene expression data, we narrowed the QTLs to eight candidate genes, including *Plcb1*, a phospholipase that is known to influence hippocampal oscillations. We also identified two genes coding for calcium channels, *Cacna1b* and *Cacna1e*, which mediate presynaptic transmitter release and have not been shown to regulate hippocampal network activity previously. Furthermore, we showed that the amplitude of the hippocampal oscillations is genetically correlated with hippocampal volume and several measures of novel environment exploration.

## Introduction

The hippocampus is critical for a wide range of emotional and cognitive behaviors. Changes in hippocampal oscillatory activity have been established during hippocampus dependent behaviors, such as anxiety-related behavior and spatial orientation [1], [2], [3]. Furthermore, an increase in amplitude of gamma oscillations in the hippocampus has been associated with memory retrieval in humans [4] and rats [5]. Together, these data suggest an important role for gamma oscillatory activity in hippocampal function.

Oscillations can be pharmacologically induced in ventral hippocampal slices of rodents by applying the acetylcholine receptor agonist carbachol [6], [7]. This *in vitro* activity, which we will refer to as “fast network oscillations”, shares many characteristics with gamma oscillations *in vivo* [8], [9]. In particular, the amplitude of *in vitro* ventral hippocampal oscillations correlates with *in vivo* gamma amplitude and performance in a memory task [10]. Moreover, we recently reported differences among eight common inbred mouse strains in traits of carbachol-induced fast network oscillations in hippocampal slices, which implies the contribution of genetic variation to these traits [11]. Therefore, *in vitro* hippocampal activity is a physiologically relevant source of information to identify genetic variants affecting hippocampal function.

Here, we aimed at identifying genes that underlie variation in hippocampal spontaneous activity and carbachol-induced oscillations *in vitro*, using a population of 29 BXD recombinant inbred mouse strains [12]. The BXD strains were derived from an intercross of the common inbred mouse strains C57BL/6J and DBA/2J, which differ in many neurophysiologic hippocampal traits and hippocampus-related behavioral traits. For example, C57BL/6J outperforms DBA/2J in spatial memory tasks [13], [14], [15], which has been associated with their differences in synaptic plasticity [16], and hippocampal mossy fiber projections [17], [18], [19]. The BXD strains, therefore, form an excellent resource to identify the segregating genetic variants that affect hippocampus-related traits, and they enabled us to identify quantitative trait loci (QTLs) associated with these traits. These QTLs contained many candidate genes and, therefore, we used gene expression data to identify genes of which the expression is linked to hippocampal activity. Using this approach, we identified three genes that were linked to hippocampal activity previously and we identified five novel candidate genes.

In addition we questioned whether genetic predisposition for having a certain level of amplitude, frequency or coherence of hippocampal activity affects behavior. To address this, we computed correlations between the hippocampal activity traits and the behavioral phenotypes assembled in the GeneNetwork database (www.genenetwork.org). We found that several behavioral traits and hippocampal activity parameters were correlated in the mouse strains used, indicating a shared genetic component.

## Results

**Figure 1:**
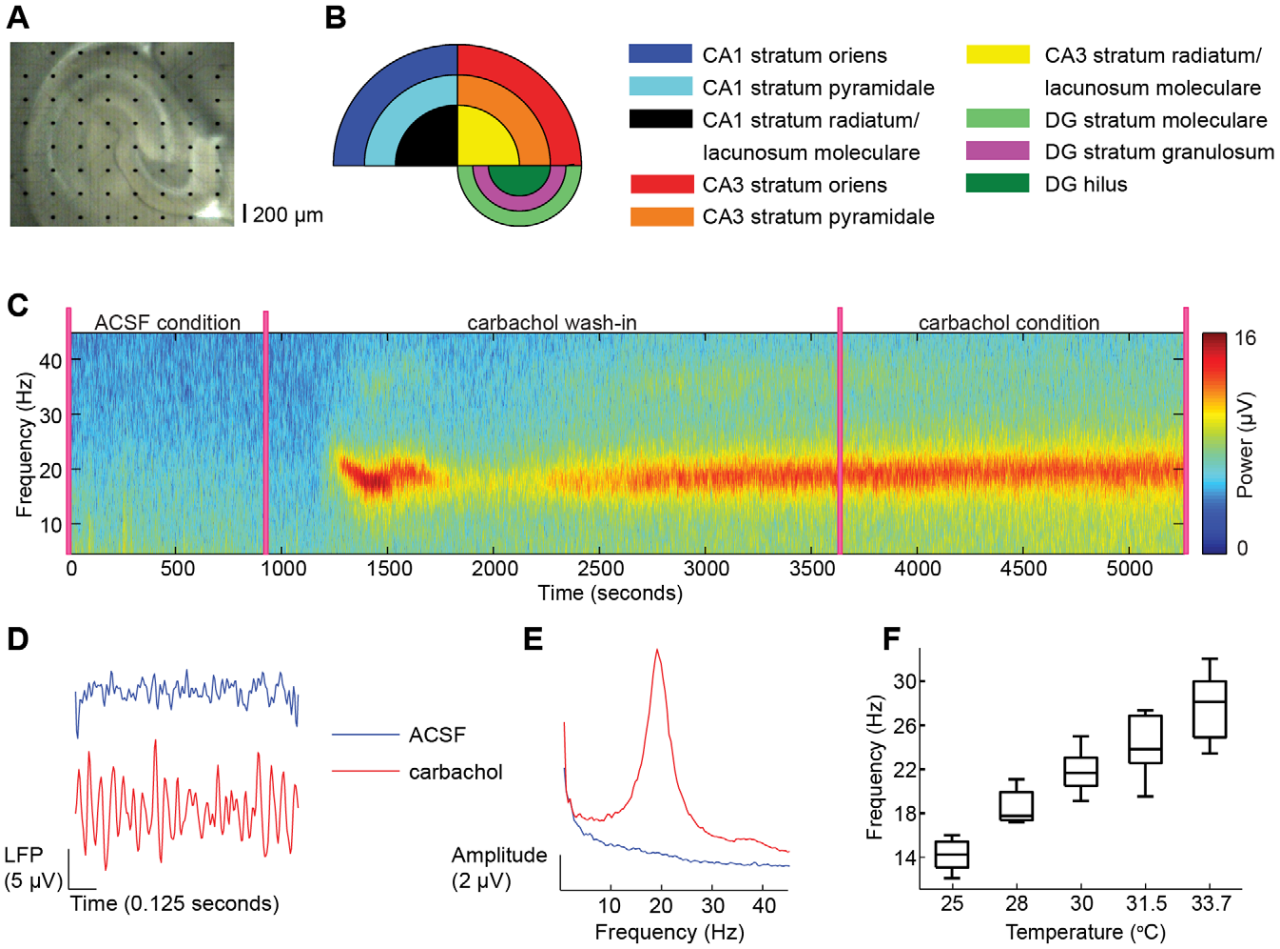
Local field potentials were recorded in hippocampal slices with multi-electrode arrays during ACSF and during application of carbachol. (A) The 8-by-8 multi-electrode array covering a slice of the mouse hippocampus. Black dots are electrodes and the spacing is 200 µm. For every slice, a photograph was taken to classify the electrode location into one of the nine hippocampal subregions shown in (B). (C) Time-frequency representation of a signal in CA3 stratum pyramidale for the complete experimental recording. (D) Examples of broadband (1–45 Hz) local field potential (LFP) traces in the ACSF (*blue*) and carbachol (*red*) condition. (E) Amplitude spectra of representative signals in each condition. (F) Box-plot summaries of peak frequencies of carbachol-induces oscillations, measured at different temperatures. The frequency approaches the gamma-frequency range (>30 Hz) at physiological temperature.

**Figure 2:**
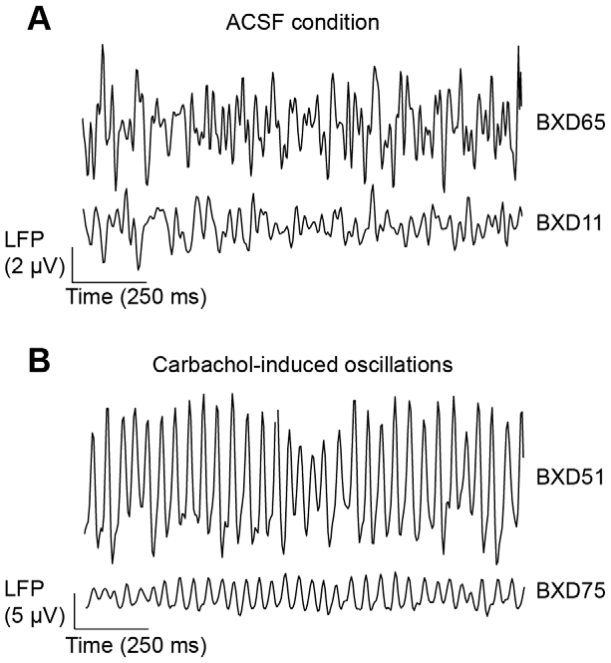
BXD mouse strains have different local field potential amplitudes. (A) Recordings from hippocampal slices in ACSF show that mouse strains differ in the amplitude of local field potential (LFP) fluctuations, as illustrated by representative traces recorded in strains with the lowest (BXD11) and highest (BXD65) amplitudes in this condition. (B) Also during carbachol-induced oscillations, we observed marked amplitude differences amongst strains; BXD51 mice had the highest, and BXD75 mice the lowest amplitudes. Depicted signals are broadband (5–40 Hz) from CA3 stratum pyramidale.

To identify genes that affect hippocampal activity, we measured local field potentials (LFPs) in hippocampal slices from 29 BXD recombinant inbred strains. Measurements were performed using 60-channel multi-electrode arrays that covered the entire hippocampal cross-section in the slice (Fig. 1A), and the electrodes were classified as located in one of nine anatomical subregions (Fig. 1B). In the first condition, slices were perfused with artificial cerebrospinal fluid (ACSF), which gave rise to asynchronous activity characterized by 1/*f-*like amplitude spectra (Fig. 1C–E). We computed the integrated amplitudes in the frequency bands 1–4, 4–7, 7–13, 13–25, 25–35, and 35–45 Hz. These amplitudes differed considerably across mouse strains as illustrated with the two extreme mouse strains in Figure 2A.

Following the ACSF condition, we applied the acetylcholine receptor agonist carbachol (25 µM) to pharmacologically induce fast network oscillations (see Fig. 1C–E and Materials and Methods). The amplitude of these oscillations also differed conspicuously between strains (Fig. 2B). To selectively analyze the effect of carbachol on hippocampal activity, we divided the value of a trait in the carbachol condition by that obtained in the ACSF condition and computed heritability scores and genetic correlations.

### Hippocampal activity traits exhibit prominent heritability and genetic correlations

**Figure 3:**
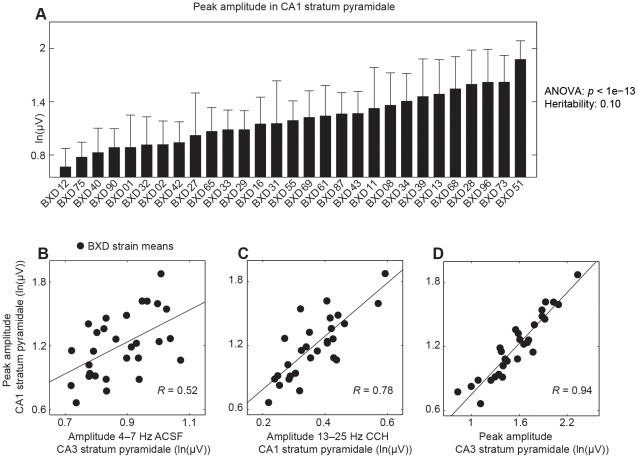
Different properties of hippocampal network activity *in vitro* depend on different genes. (A) The mean and SEM of peak amplitude in the CA1 stratum pyramidale for each of the 29 BXD strains. The heritability indicates that the peak amplitude is sensitive to genetic variation of the BXD strains. (B–D) Scatter plots of trait means per BXD strain of peak amplitude in CA1 stratum pyramidale versus (B) the integrated amplitude at 4–7 Hz during the ACSF condition in CA3 stratum pyramidale, (C) the integrated amplitude at 13–25 Hz in CA1 stratum pyramidale, and (D) the peak amplitude in CA3 stratum pyramidale. The correlation of the strain means is an estimate of the genetic correlation, which ranged from low (B) to high (D), indicating different and similar underlying genes, respectively.

The analysis of amplitude, peak frequency and inter-areal correlations (see Materials and Methods) for the two conditions in the nine hippocampal subregions define a total of 198 trait values per slice. Several traits were observed to exhibit prominent variation across the mouse strains, e.g., the peak amplitude in the presence of carbachol varied by a factor of three (Fig. 3A) in the CA1 stratum pyramidale. *P*-values from *F* statistics (ANOVA) and heritability scores were calculated for every trait (Tables S1 and S2). The heritabilities ranged from 1 to 25%.

Interestingly, we observed a wide range of genetic correlations between the 198 traits, as illustrated by the scatter plots in Figure 3B–D. A low genetic correlation indicates that traits are influenced by different genes, whereas a high genetic correlation suggests that traits have the same underlying genes.

**Figure 4:**
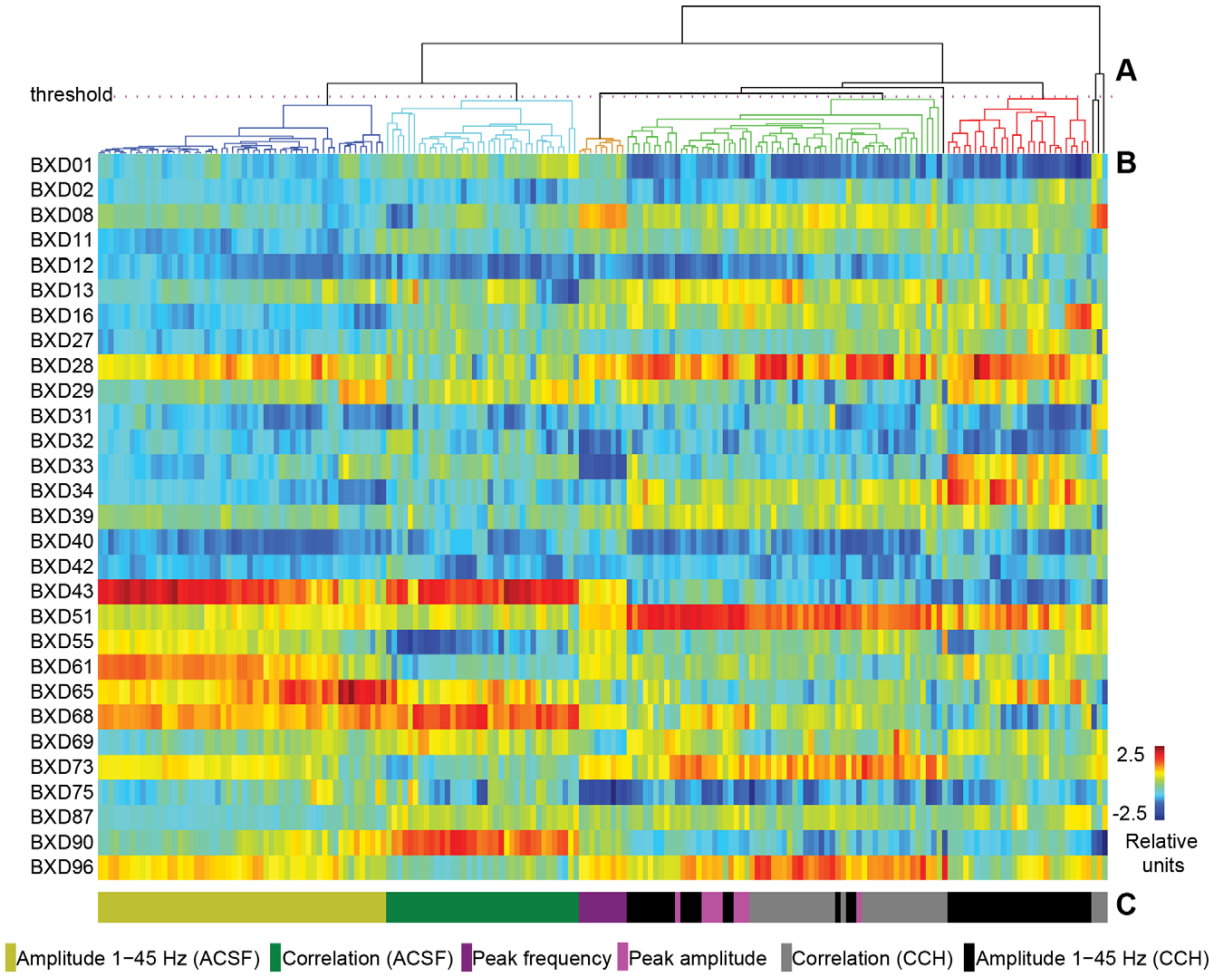
Clusters of genetically correlated traits correspond to distinct experimental conditions and properties of oscillations. The 198 traits (columns) for each of the 29 mouse strains (rows) were clustered according to their genetic correlation. (A) A threshold of 0.45 has been introduced to mark the major clusters in the dendrogram. (B) The mean trait value is represented in color code for each strain after normalization across strains, i.e., for every column the mean equals zero and the variance equals one. The clustering in (A) largely corresponds to six classes of traits as indicated by the color-coding in (C), i.e., the peak frequency and peak amplitude for the carbachol (CCH) condition, and the broad-band amplitude (1–45 Hz) and the inter-regional correlations for both conditions. We based the QTL mapping on the mean of the traits within these six classes (see labeling below the cluster diagram).

We performed a cluster analysis to evaluate the genetic correlation structure of the set of 198 traits (Fig. 4). The distance measure between traits reflected genetic correlation. The number of clusters depends on the threshold for the minimal distance between the clusters (see Materials and Methods). A threshold of 0.45, i.e., allowing for a maximal mean correlation between clusters of 0.55, resulted in clusters that largely correspond to six main classes representing experimental conditions and type of analyses (Fig. 4A–B). For the ACSF condition, one class contains all the interregional correlations (*n* = 36), and one class the amplitudes in the nine regions from all frequency bands (1–4, 4–7, 7–13, 13–25, 25–35, 35–45 Hz, *n* = 54). For the carbachol condition, the four classes contain the phase-locking factors (*n* = 36), the amplitudes from all frequency bands and subregions (*n* = 54), the peak frequencies (*n* = 9) and the peak amplitudes (*n* = 9), respectively. We color-coded each of the 198 traits according to this classification in Figure 4C, to visualize performance of the clustering analysis in separating the six main classes of traits. Because the traits were strongly correlated within the six classes, for each class we calculated the mean over the strain means (per strain) for QTL analysis. From this point onward, the number of traits was reduced to six, and they will be referred to as: correlation (ACSF) (GeneNetwork ID 13484), amplitude 1–45 Hz (ACSF) (ID 13486), correlation (CCH) (ID 13491), amplitude 1–45 Hz (CCH) (ID 13490), peak amplitude (ID 13487) and peak frequency (ID 13488).

### Amplitude of carbachol-induced oscillations shows prominent genetic correlation with hippocampal volume and locomotion traits

Studying hippocampal activity in BXD strains opens up the exciting possibility to relate genetic variation in brain activity to that of phenotypes from the GeneNetwork database, which contains more than 2000 behavioral, anatomical and physiological traits from previous studies on BXD strains. We computed genetic correlations between the hippocampal activity traits and two subsets of phenotypes from the GeneNetwork database (Materials and Methods). See Tables S3 and S4 for descriptions of phenotypes in the subsets.

**Figure 5:**
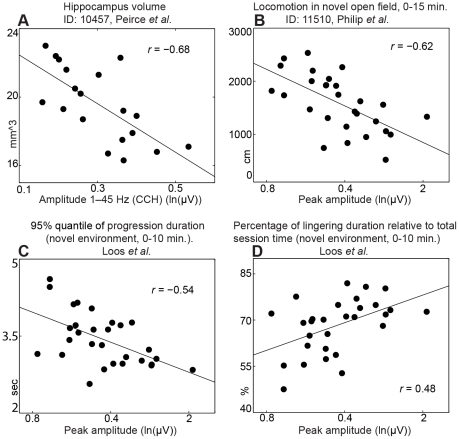
Amplitude of carbachol-induced oscillations shows prominent genetic correlations with hippocampal volume and locomotion traits. Scatter plots of (A) amplitude 1–45 Hz versus hippocampus volume, and (B) peak amplitude versus locomotion in novel open field. More detailed analysis of locomotion revealed a negative correlation with the duration of progression segments (C), but a positive correlation with the duration of lingering segments (D). The black dots represent BXD strain means. IDs refer to the GeneNetwork database.

The first subset (*n* = 35) consisted of physiological traits of the hippocampus, such as the weight or volume of different subregions of the hippocampus. Interestingly, the trait amplitude 1–45 Hz (CCH) was negatively correlated with volume of the hippocampus. The four phenotypes from the subset with the most significant correlations with amplitude 1–45 Hz (CCH), were two measures of hippocampus volume (GeneNetwork ID 10457: *r* = −0.68, *p*<0.002 (Fig. 5A) and ID 10456: *r* = −0.66, *p*<0.002 [20]), and two measures of ventral hippocampus volume (ID 10756: *r* = −0.57, *p*<0.01 and ID 10757: *r* = −0.53, *p*<0.05 [21], uncorrected *p*-values). The four correlations were significant at a false discovery rate of 0.125.

The second subset (*n* = 351) consists of a selection of behavioral traits from the database (see Materials and Methods). We found strong negative correlations between peak amplitude and four traits representing locomotion in a novel environment (ID 11510: *r* = −0.62, *p*<0.0005 (Fig. 5B), ID 10916: *r* = −0.83, *p*<0.0005, ID 10037: *r* = −0.76, *p*<0.001, ID 10416: *r* = −0.89, *p*<0.005, uncorrected *p*-values). The four locomotion traits were strongly correlated with each other, despite having been measured in different studies [22], [23], [24], [25], which reflects that locomotion is a very reproducible trait [26]. The four locomotion traits were part of the top-10 of strongest correlations, which were all significant at a false discovery rate of 0.125. Interestingly, we also found a high positive correlation of peak amplitude with the performance in the Morris water maze task [27] (ID 10816, *n* = 7, *r* = 0.74, *p*<0.05, uncorrected *p*-value). This correlation, however, did not survive correction for multiple testing, possibly because of the low number of observations.

We then measured locomotion in a novel open field in several BXD strains in our own laboratory. We used SEE software to dissect locomotion into lingering and progression segments (see Materials and Methods). Peak amplitude correlated negatively with total distance moved (*r* = −0.52, *p*<0.005, data not shown) as it was found also using the GeneNetwork database (Fig. 5B), and with the duration of progression segments (*r* = −0.54, *p*<0.005, Fig. 5C), but positively with the duration of lingering segments (*r* = 0.48, *p*<0.01, Fig. 5D). Taken together, these findings indicate that the inverse relation between peak amplitude and locomotion in a novel environment is a robust effect.

### QTL mapping identifies shared and unique genetic influences on hippocampal traits

We used the six main traits, as derived from the cluster analysis (Fig. 4C), for QTL mapping (see Materials and Methods). In total, we identified two significant QTLs (*p*<0.05) and seventeen suggestive QTLs (*p*<0.63) (Fig. 6). In Figures S1, S2, S3, S4, S5, S6, S7, S8, S9, S10, S11, S12, S13, S14, S15, S16, S17, S18, S19 close-ups of the QTLs are shown. See Tables S5, S6, S7, S8, S9, S10, S11, S12, S13, S14, S15, S16, S17, S18, S19, S20, S21, S22, S23 for the location of the nineteen QTLs and the genes they contain. Table S24 contains the locations of all QTL intervals.

**Figure 6:**
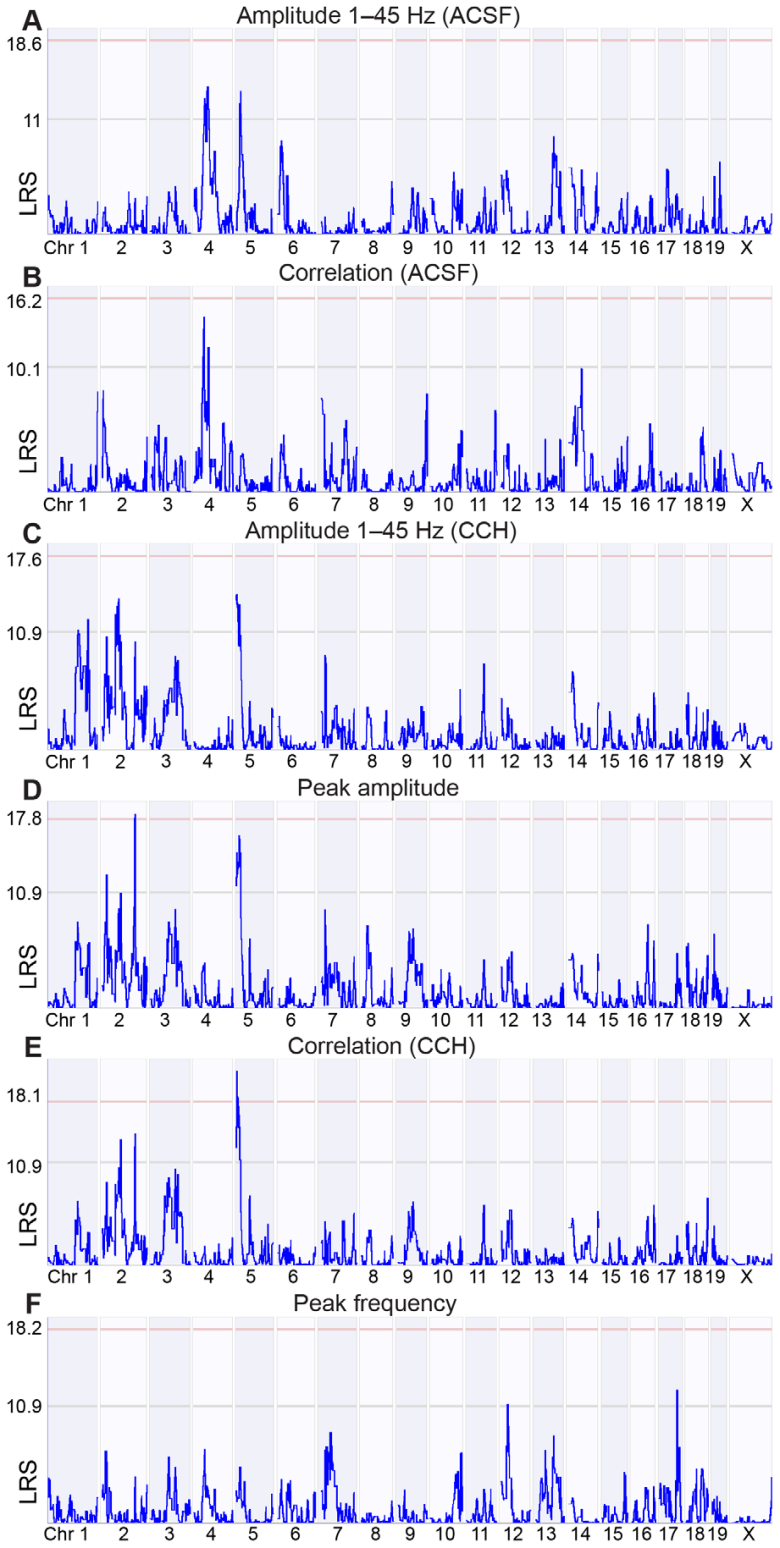
QTL mapping of six hippocampal activity traits peaks at 19 different locations. The LRS scores (*y*-axis) quantify the relation between genomic markers (*x*-axis) and six traits. Integrated amplitude between 1 and 45 Hz at the ACSF (A) and carbachol (CCH) condition (C), peak amplitude (D) and frequency (F) at the carbachol condition, inter-regional correlation at the ACSF (B) and carbachol condition (E). The red horizontal lines indicate the threshold for significance (*p* = 0.05), whereas the grey lines indicate suggestive significance (*p* = 0.63). For the traits depicted here, we selected the 18 QTLs above the suggestive significance level and correlated the hippocampal traits with expression data of genes within these QTLs.

Amplitude 1–45 Hz (ACSF) and correlation (ACSF) had overlapping QTLs located on chromosome four (Fig. 6A, B). Amplitude 1–45 Hz (CCH), peak amplitude, and correlation (CCH) had overlapping QTLs on chromosome five; the one from peak amplitude overlapped a QTL from Amplitude 1–45 Hz (ACSF) (Fig. 6A, C–E and Fig. S3, S11, S14 and S17). Also, we identified for each trait one or more suggestive QTLs that were not found for other traits. The partially shared QTLs suggest that the traits share genetic components in addition to having unique genetic component(s). For example, peak frequency (Fig. 6F) had no QTLs in common with other traits. This suggests a dissimilar genetic underpinning of peak frequency and, e.g., peak amplitude.

### Correlation with gene expression data points to candidate genes

The nineteen suggestive or significant QTLs identified (see above) varied in length from 2 to 19 Mb, and contained between 6 and 155 genes each. In order to evaluate these genes, we correlated the hippocampal activity traits with expression data from the hippocampus of BXD mice (see Materials and Methods).

For each of the six main traits, we selected genes within the QTLs of the trait, and correlated the expression of these genes with the trait. The significance of the correlations was determined with permutation tests (see Materials and Methods). Table 1 gives an overview of the eight genes from these nineteen QTLs that had significant expression correlations. Peak amplitude was associated with *Plcb1* (phospholipase C, beta 1) and *Cacna1b*, the gene coding for calcium channel alpha1B. The gene coding for calcium channel alpha1E (*Cacna1e*) was linked to amplitude 1–45 Hz (CCH). *Plcb1* is known to influence hippocampal oscillations [28]. *Cacna1b* and *Cacna1e* have been implicated in hippocampal LTP [29], [30], but not in the formation of synchronous network activity. For peak frequency, we identified Eps15-homology domain protein 3 (*Ehd3*), which, like the other genes identified (*Creb3, Psmc2*, *Dctn3*, and *Ralgps2*) has not yet been related to hippocampal activity.

**Table 1:** QTL mapping and correlation with gene expression revealed eight candidate genes for influencing hippocampal activity.

trait	location QTL	LRS	genes	Correlation	*p*-value
Amplitude 1–45 Hz (ACSF)	chr 5 16.522–22.723	14.6	Psmc2	−0.63	0.01
Correlation (ACSF)	chr 4 39.167–44.254	15.1	Dctn3	−0.59	0.04
			Creb3	−0.59	0.04
Amplitude 1–45 Hz (CCH)	chr 1 156.371–160.384	12.4	Ralgps2	0.61	0.03
			Cacna1e	0.62	0.01
Peak amplitude	chr 2 132.507–135.784	18.2	Plcb1	0.58	0.02
	chr 2 22.673–25.693	12.2	Cacna1b	0.57	0.03
Peak frequency	chr17 72.002–76.447	12.6	Ehd3	0.60	0.005

Each row contains the information belonging to one candidate gene. Indicated are the hippocampal activity trait associated with the gene, the location of the QTL (chr = chromosome, location in Megabases) that harbors the gene found, the LRS score of the QTL, the gene symbol, the correlation between trait and level of expression of the gene, and finally the *p*-value from the correlation, computed with a permutation test.

## Discussion

Neuronal oscillations have been implicated in cognitive and emotional behavior [1], [31], [32] and are heritable [11], [33], [34] which make quantitative traits derived from oscillatory activity potentially useful in gene-finding strategies. Here, we searched for genes that underlie variation in hippocampal network activity *in vitro* based on 29 recombinant inbred strains from the BXD population [35]. QTL mapping pointed to regions on the genome associated with variability in amplitude of oscillatory and non-oscillatory activity, as well as in functional coupling between hippocampal areas. To evaluate genes in the QTLs for a potential contribution in hippocampal activity, we correlated their expression in the hippocampus with the hippocampal activity traits, and identified eight candidate genes.

### Hippocampal activity traits have relatively low heritability in BXD strains

The heritability estimates of amplitude and functional coupling ranged from 1 to 25%, which is similar to what we found in a population of eight inbred mouse strains [11]. Higher-order statistical measures of oscillatory dynamics, such as long-range temporal correlations [36] and markers from Langevin dynamics [37] exhibit low—albeit significant—heritability, and were not included in the present QTL analysis [38].

To our knowledge, heritability of *in vivo* hippocampal gamma-band amplitude has not been estimated yet, but EEG studies in humans show that the early auditory gamma-band response has a heritability of 65% [39], and heritability of amplitude in the classical delta-, theta-, alpha- and beta-frequency bands ranges from 40 to 90% [33]. Thus, the heritability we observed here may be considered low. This may be explained by the environmental noise introduced by the experimental procedure, e.g., the slicing of the hippocampus. Moreover, heritability depends also on the population in which it is measured; the heritability we estimated holds for the offspring of the strains C57BL/6J and DBA/2J, which obviously does not comprise the genetic variation as present in the human population.

### Reduction of traits inspired by cluster analysis

We used cluster analysis to evaluate the genetic correlations of the 198 hippocampal activity traits. The clusters showed which traits are strongly correlated and, therefore, could be merged. The clusters we identified exhibited a great overlap with six main classes of traits representing experimental conditions and type of analyses. We chose to supervise the merging of traits by using the classes instead of the exact clusters. This approach had the advantage over commonly used unsupervised methods, such as principal component analysis, that the resulting traits have a straightforward analytic and physiological interpretation. By collapsing the information on hippocampal subregions and frequency bands, we reduced the amount of traits to six. The cluster analysis showed that the genetic correlation between the traits measured during the ACSF condition and those during the CCH condition is relatively low. The QTL mapping, however, showed that this correlation is substantial: the traits from the ACSF condition have some overlapping and some non-overlapping QTLs with the traits from the carbachol condition, suggesting a partially unique and partially shared genetic architecture. Therefore, it is also likely that partially shared and partially unique downstream mechanisms underlie the traits from the two conditions.”

### Genetic correlations with behavioral traits from the GeneNetwork

The negative genetic correlation between hippocampal volume and hippocampal activity traits suggests that there are genes that influence both traits. In two subsequent BXD studies [20], [40], a QTL for hippocampal volume was reported at chromosome 1, which overlaps with one of the QTLs we identified for amplitude 1–45 Hz (CCH). This QTL might contain genes that influence both hippocampal activity and hippocampal volume. Recently it has been reported that tenascin-C deficient mice have smaller hippocampal subregions and higher gamma oscillation amplitude compared to wild-type mice [41], which corroborates our finding that small hippocampal volume is associated with high amplitude oscillations.

Locomotion in a novel open field is a complex trait used as a measure for, e.g., exploration, anxiety and hyperactivity. The locomotor behavior of a mouse that is placed in a novel environment can be divided in lingering and progressing segments [42]. During lingering, the animal is actively gathering information about the environment by sniffing, rearing and looking around. During progression, the animal moves from one location to the next. We observed that peak amplitude was negatively correlated with the duration of the progression segments, but positively with the duration of the lingering segments. Future studies should test whether the same relation holds between locomotion and network oscillations in freely behaving mice. This is not unlikely, because hippocampal oscillations in the 20–40 Hz range are prominent when mice enter a novel environment [43], and gamma oscillations have been associated with novelty in rats [44].

The positive correlation between the performance in the Morris water maze and the peak amplitude suggests that BXD strains capable of producing high-amplitude gamma have good spatial memory. Elevated activity of gamma oscillations during encoding and retention of information in working memory has been reported in humans [4], [45] and in rodents [5] Our results, however, provide the first indication that genetic predisposition for high-amplitude gamma oscillations is beneficial for working-memory performance.

### Genes previously associated with carbachol-induced hippocampal oscillations

Genetic influences on hippocampal carbachol-induced oscillations *in vitro* have been studied extensively and it has pointed to several genes involved, including *Chrm1* [46], *Gabra5* [47], *Gabrb2* [48], *Plcb1* [28]. *Plcb1* is essential for the genesis of carbachol-induced oscillations as indicated by the inability to induce oscillations with carbachol in the hippocampus of *Plcb1* knockout mice [28]. *Plcb1* is one of the candidate genes we identified, which can be regarded as an internal validation of our experimental and statistical procedures.

Our paradigm did not reveal other genes previously associated with hippocampal oscillations. A reason for this may be that the influence of such a gene may be caused by only a few single-nucleotide polymorphisms (SNPs). If C57BL/6J and DBA/2J do not differ in these SNPs, the paradigm we followed would not have revealed these genes. Moreover, most of the studies that try to link genes to brain activity use knockout-mice, in which the effect of the particular gene is likely to be stronger than in the BXD population. Also, the effect sizes of the genes known to be involved in hippocampal oscillations may be too small to be detected by our analysis.

### Novel candidate genes associated with hippocampal activity

Our combined use of QTL mapping and correlation with expression data has some notable advantages. The QTL mapping was merely used to select stretches of the genome for further analysis, which justifies the use of suggestive significance. We qualified our findings with the significance level of the correlation with the expression data of genes within the QTLs. This significance increases because of the use of the relatively small QTLs.

We identified two candidate genes for shaping hippocampal network that code for calcium channels: the alpha1b subunit (*Cacna1b*), and the alpha1e subunit (*Cacna1e*). Calcium channels mediate synaptic transmission [49], and are essential in the formation of thalamo-cortical gamma band activity [50]. Also, *Cacna1e* and *Cacna1b* facilitate hippocampal long-term potentiation (LTP) [29], [30], and the *Cacna1b* knock-out mouse exhibits impaired long-term memory and LTP [51]. Thus, *Cacna1e* and *Cacna1b* are interesting candidates for playing a role in hippocampal oscillations. Moreover, *Cacna1b* has been associated with schizophrenia in three recent linkage studies [52], [53], [54]. Thus, we may hypothesize that alterations in *Cacna1e* and *Cacna1b* affect hippocampal network activity such as to impair memory performance in, for example, schizophrenia patients known to suffer from memory impairment.

In a QTL for correlations (ACSF) we identified the gene *Creb3*, coding for the transcription factor cAMP responsive element-binding protein 3. *Creb1* plays an important role in (spatial) memory [55]; increasing the expression level of *Creb1* in the hippocampus facilitates long-term memory [56]. Therefore, it might well be that *Creb3* is involved in hippocampal activity as well. The other gene identified for this trait is *Dctn3*, which has a function in the cytoskeleton [57]. Peak frequency was linked to *Ehd3* which is involved in endosome to Golgi transport [58]. *Psmc2*, associated with amplitude 1–45 Hz (ACSF), is involved in developmentally programmed cell death [59]. *Ralgps2*, linked to amplitude 1–45 Hz (CCH), affects neurite outgrowth [60].

In summary, we identified eight candidate genes for influencing different aspects of hippocampal network activity. Future research, by means of knockout mice or pharmacological manipulations, should reveal the mechanisms by which these genes affect hippocampal activity and related cognitive functions.

## Materials and Methods

### Animals, hippocampal slice preparation and extracellular recording

All experiments were performed in accordance with the guidelines and under approval of the Animal Welfare Committee of the VU University Amsterdam. BXD strains were originally received from Jackson Lab, or from Oak Ridge Laboratory (BXD43, BXD51, BXD61, BXD65, BXD68, BXD69, BXD73, BXD75, BXD87, BXD90), and were bred by the NeuroBsik consortium. In this study we used in total 586 slices from 322 animals (62% male), from 29 BXD strains: BXD01 (*n* = 20), BXD02 (*n* = 28), BXD08 (*n* = 18), BXD11 (*n* = 12), BXD12 (*n* = 20), BXD13 (*n* = 16), BXD16 (*n* = 22), BXD27 (*n* = 13), BXD28 (*n* = 13), BXD29 (*n* = 8), BXD31 (*n* = 14), BXD32 (*n* = 23), BXD33 (*n* = 18), BXD34 (*n* = 24), BXD39 (*n* = 17), BXD40 (*n* = 18), BXD42 (*n* = 28), BXD43 (*n* = 17), BXD51 (*n* = 36), BXD55 (*n* = 22), BXD61 (*n* = 16), BXD65 (*n* = 18), BXD68 (*n* = 20), BXD69 (*n* = 28), BXD73 (*n* = 27), BXD75 (*n* = 23), BXD87 (*n* = 31), BXD90 (*n* = 19), and BXD96 (*n* = 17). Per animal, maximally 2 slices were used. Unanaesthetized mice were decapitated at postnatal day 13–15. The brains were quickly dissected and placed in ice-cold artificial cerebrospinal fluid (ACSF) containing 125 mM NaCl, 25 mM NaHCO_3_, 3 mM KCl, 1.2 mM NaH_2_PO_4_, 1 mM CaCl_2_, 3 mM MgSO_4_, and 10 mM D(+)-glucose (carboxygenated with 5% CO_2_/95% O_2_). Horizontal slices (400 µm thick) from the ventral hippocampus were cut by a microtome (Microm, Waldorf, Germany). Slices were stored in an interface storage chamber at room temperature and placed in ACSF containing 2 mM CaCl_2_ and 2 mM MgSO_4_. After 1 hour, slices were placed on 8-by-8 planar electrode grids with 200 µm spacing between electrodes (the 4 corners of the grid did not contain electrodes; see Fig. 1A) and polyethylenimine coating (Sigma, St. Louis, MO, USA). The slices were left for 1 hour in a chamber with humidified carbogen gas before they were placed in the recording unit. During recordings the flow rate was 4–5 ml/min and the temperature was kept at 30±0.3°C. Carbachol was purchased from Sigma. Local field potentials (LFPs) were measured at each of the 60 electrodes, sampled at 1 kHz, down-sampled off-line to 200 Hz and converted into Matlab (The Mathworks, USA) file format. Off-line analysis was done using custom written scripts in Matlab.

### Slice selection and subregion classification

For each experiment a photograph was taken of the slice in the recording unit, to visualize the locations of the electrodes in the hippocampus (Fig. 1A). The hippocampus consists of three main anatomical regions: CA1, CA3 and dentate gyrus (DG). We divided CA3 and CA1 into the subregions stratum oriens, stratum pyramidale and stratum radiatum/lacunosum-moleculare, and DG into stratum moleculare, stratum granulosum and hilus (Fig. 1B). To classify electrode locations into one of these nine subregions, we used an in-house written interactive Matlab procedure based on the photograph of the electrode grid. Using Fourier analysis (see below), we determined for each electrode whether oscillatory activity was present. A slice was excluded from further analysis if none of the 60 electrodes showed oscillations.

For each condition, in order to detect electrodes producing noisy signals and transient artifacts before the quantitative trait analysis, each slice recording was subjected to a principal component analysis. If noisy signals were present, then the first few spatial components had high values only for one or a few of these signals. These signals were identified and excluded. The time series of the remaining signals were averaged; this average was used to identify noisy segments. Samples from this average with absolute values exceeding five times the standard deviation of the averaged signal, were excluded from each signal before the analysis.

### Experimental protocol to measure hippocampal network activity

After placing the slices in the recording units with ACSF, 15 minutes of spontaneous activity was recorded (see Fig. 1C). These first 15 minutes will be referred to as the “ACSF condition”. Then, carbachol (25 µM) was bath applied to the slice. Carbachol-induced oscillations at around 20 Hz were initially unstable in frequency and amplitude, but stabilized after 45 minutes. After this 45-minute wash-in period fast network oscillations were recorded for a period of 30 minutes, which will be referred to as the “carbachol condition”. In Figure 1C a time-frequency representation of a representative signal is shown for a complete recording. Example LFP traces for the two conditions are shown in Figure 1D. The frequency of oscillations increased with temperature (Fig. 1F), which has been observed previously [61], [62]. Thus, the oscillations at around 20 Hz, which were recorded at 30°C in the present experiments, are expected to have frequencies in the gamma range (>30 Hz) at the physiological temperature of 36.9°C. However, the amplitude of oscillations at higher temperatures was markedly lower than at 30°C, resulting in an unfavorable signal-to-noise ratio. Therefore, all experiments were performed at 30°C.

### Fourier analysis

For the two conditions (ACSF and carbachol), and for each electrode that was classified into one of the nine regions, we calculated the Fourier amplitude spectrum using Welch's method [63]. Figure 1E shows representative spectra in the two conditions. For the ACSF condition, we calculated the integrated amplitude in the frequency bands 1–4, 4–7, 7–13, 13–25, 25–35, and 35–45 Hz. In the carbachol condition, we observed oscillations at around 20 Hz, which is similar to previous reports using a temperature of around 30°C in mouse hippocampus [11], [64], [65]. We calculated the amplitude and the frequency of these oscillations, which we will refer to as the peak amplitude and the peak frequency, respectively. Moreover, a 1/*f* curve was fitted to the spectrum outside the interval at which the peak occurred, and from this curve we calculated the integrated amplitude in the frequency bands 1–4, 4–7, 7–13, 13–25, 25–35, and 35–45 Hz. For each of these measures, the traits we used for the cluster analysis (see below) were the mean trait values across electrodes per anatomical subregion (*n* = 54 traits for the ACSF condition, *n* = 72 traits for the carbachol condition).

To establish whether oscillations were detected at a given electrode, we applied the following procedure. First, a frequency interval in which the peak of the spectrum occurred was determined visually, e.g., for the spectrum in Figure 1E this interval would be from 10 to 25 Hz. Next, a 1/*f* curve was fitted to the spectrum outside this interval. This 1*/f* curve was then subtracted from the original spectrum. Finally, a Gaussian curve was fitted to the remaining spectrum. If the peak of this Gaussian curve did not exceed the 95% confidence interval of the fitted 1*/f* curve, we classified the signal as not oscillating. Slices were excluded from further analysis when none of the electrodes detected oscillations.

### Interaction between hippocampal regions

To quantify the interaction between two hippocampal subregions, e.g., between CA1 stratum oriens and CA3 stratum oriens, we calculated a suitable cross-correlation measure (as described below) between signals from all possible pairs of electrodes from these subregions, and used the mean over these pairs for the cluster analysis (see below).

Oscillatory activity was not observed in the ACSF condition and, therefore, we quantified cross-correlations between subregions in this condition using Pearson's linear correlation of the LFPs. Prior to this analysis, the signals were filtered between 5 and 40 Hz to remove the fairly large amount of noise outside this interval. Thus, for every pair of subregions, the mean correlation over all possible electrode-pairs from the subregion-pair was used (*n* = 36 traits).

In the carbachol condition, in contrast, the signals were strongly oscillatory. Therefore, we calculated the phase-locking factor (PLF) between signals in this condition. The PLF is a well established measure for quantifying the interaction between two oscillating signals that can be out of phase and possibly have independent amplitude fluctuations [66], [67]. To reduce volume conduction effects, the current-source density of the LFPs was computed [9], [68]. After this transformation, we computed the phase-locking factors between signals that were band-pass filtered 4 Hz around the peak frequency of the fast network oscillations, for every subregion pair (*n* = 36 traits).

### Normalization

To specifically analyze the effect of carbachol, we normalized the amplitude and correlation traits of carbachol-induced oscillations by dividing them by the same traits from the ACSF condition, except for the peak frequency, because there was no peak in the amplitude spectrum during ACSF. For the same reason, the peak amplitude from the carbachol condition was normalized by the integrated amplitude between 15 and 25 Hz from the ACSF condition. The PLF traits from the carbachol condition were divided by the correlation traits from the ACSF condition. Thus, the normalized traits express the relative sensitivity to experimental manipulations.

### ANOVA

To determine whether a given trait differed significantly between mouse strains, we performed a one-way ANOVA and the corresponding *F*-test with the trait as dependent variable and the mouse strain as factor. The null hypothesis of this test is that for at least one strain the trait mean is significantly different from the trait means of the other strains. Where necessary, the data were transformed with the natural logarithm order not to violate the normality assumption for ANOVA.

### Heritability

The observed value of a trait (e.g. peak amplitude) from a given slice is the result of both genetic and environmental influences, including measurement noise. To quantify the extent to which a trait is influenced by genetic factors, we computed its heritability. The heritability of a trait is a measure for the proportion of the total variance of the trait that is caused by genetic variation. The remainder of the variance is assumed to be due to environmental factors. For inbred strains the heritability *h* ^2^ of a trait can be defined as 
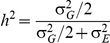
, where σ^2^ *_G_* is the component of variance between strains, and σ ^2^ *_E_* the component of variance within strains [69]. The value of *h* ^2^ ranges between 0 and 1, where 0 means no genetic contribution to the trait, and 1 means that the trait is controlled only by genetic factors. We estimated heritability as described in detail in Jansen *et al.* (2009).

### Genetic correlation between traits

To reveal the extent to which two traits share genetic factors, we studied the correlation between the genetic effects of the two traits, the so-called genetic correlation. For inbred strains, we can estimate the genetic correlation between two traits as the Pearson's linear correlation between the 29 mouse strain means of one trait and the 29 mouse strain means of the other trait [69], [70]. The mouse strain means were taken over all slices from a given mouse strain. The estimated genetic correlations were used in a cluster analysis, as explained below.

### Cluster analysis of traits

In order to identify clusters of genetically correlated traits, hierarchical clustering was performed on the complete set of *n* = 198 traits. In this analysis, traits are clustered based on a distance measure between the traits. To measure the distance between two traits, we subtracted the estimated genetic correlation between the two traits from 1, so traits with high genetic correlation are close to each other. No strong negative correlations were present: using absolute genetic correlation yielded similar results. Average linkage was used as a clustering method. This method starts with as many clusters as there are traits, and then sequentially joins the two clusters that are closest to each other in terms of the mean of distances between all possible pairs of traits in the two clusters; the procedure ends when all traits are joined in one cluster. A particular classification of traits into clusters is obtained by setting a threshold for the minimal distance that the clusters are allowed to have between them. The result of the cluster analysis was visualized in a dendrogram, in which the sequential union of clusters was depicted together with the distance value (the height of the horizontal lines that connect the objects or clusters) leading to this union. The threshold procedure can be visualized by a horizontal line in the dendrogram; the clusters under this line correspond to that particular threshold.

### BXD recombinant inbred strains and QTL mapping

The BXD strains were created by crossing the inbred mouse strains C57BL/6J and DBA/2J and by inbreeding several groups of the crossed offspring [35]. It is one of the largest mammalian recombinant inbred strain panels currently available. Genetically, each of these BXD strains is a unique combination of the C57BL/6J and DBA/2J strains. The chromosomes of the BXD strains consist of haplotypes (stretches of chromosomes inherited intact from the parental strains). Each BXD strain was genotyped at 3795 markers covering the entire genome; each marker was classified as originating from C57BL/6J or DBA/2J. In order to compute the correlation between a trait and these markers, the markers were encoded, −1 for DBA/2J version of the marker and 1 for C57BL/6J version of the marker. Markers that correlate with a trait are called QTLs. We used WebQTL (www.genenetwork.org) to compute and visualize the QTL interval mapping. In WebQTL, the correlation between a marker and a trait was transformed into likelihood ratio statistics (LRS) in the following way: LRS = N*log(1/(1-*r* ^2^)), where *N* is the number of strains, and *r* the correlation [71]. For intervals with unknown genotype, LRS scores of flanking markers were linearly interpolated. Threshold for significant LRS scores were computed using a permutation test: the *N* strain means from the trait were permuted, and for this permutation the maximum LRS score over all markers was computed, which resulted in an observation of the null-distribution. Significance of LRS scores was computed by comparing them with the empirical null-distribution. LRS scores were termed significant if *p*<0.05, and suggestive if *p*<0.63. The QTL mapping was used to select regions of the genome for further analysis, which justifies the use of suggestive significance. The QTL intervals were determined with the 1 LOD drop-off method [72]; the interval ends where the LRS score drops more than 4.61 LRS ( = 1 LOD) with respect to the maximum LRS score in the interval. As in previous studies using BXD strains [73], [74], we did not use the parental strains for QTL mapping.

### Correlations with traits from the GeneNetwork phenotype database

The GeneNetwork database (www.genenetwork.org) contains more than 2000 phenotypes from previous studies using BXD strains. We computed genetic correlations between the hippocampal activity traits and two subsets of phenotypes from this database. By using subsets, the correction for multiple testing is reduced. To further reduce the risk of chance correlations, we only included phenotypes from the database that were reported for more than six BXD strains that were also used in the present study. The first subset (*n* = 35) contained physiological traits of the hippocampus. The second subset (*n* = 351) contained the behavioral traits that do not involve pharmacological manipulations. See Tables S1 and S2 for trait description and GeneNetwork IDs of both subsets.

To correct the significance for multiple testing, we used the false discovery rate (FDR) [75], [76]. The FDR controls the expected proportion of erroneously rejected hypothesis. It is the number of falsely rejected hypotheses divided by the total number of rejected hypotheses. In our case, the total number of rejected hypotheses is the number of observed correlations with *p*-values lower than a threshold. The number of falsely rejected hypotheses was estimated with a permutation paradigm. The hippocampal activity trait was permuted thousand times across strains, and the correlation between the permuted trait and the traits from the subsets was computed. The number of falsely rejected hypotheses was estimated as the average number of correlations with *p*-values smaller than the threshold.

### Gene expression data

Data on gene expression in hippocampal tissue of adult mice, measured with Affymetrix Mouse Exon 1.0 ST Arrays, were accessed through GeneNetwork (UMUTAffy Hippocampus Exon (Feb09) RMA, accession number GN206, from www.genenetwork.org). The original data set contained over 1.2 million probe sets at exon level uniformly spread over the entire genome. Each probe set consisted of the RMA-summarized [77] value of the collective probes each targeting 25 base pairs, measured at adult mice from BXD strains [78]. For our analysis, we removed data from probe sets targeting regions that contain SNPs that differed between the two parental strains (according to databases snp_celera_b37 and snp_perlegen_b37 (2008) downloaded from http://phenome.jax.org). Probe sets targeting introns and intergenic regions were also removed, which reduced the amount of probe sets to 340318. We analyzed the expression per gene by taking the mean over all probes that target the same gene. For each hippocampal activity trait, we only calculated correlations with expression of genes from the QTLs of the trait. Significance levels for these correlations were determined with a permutation test; the hippocampal activity trait was permuted across strains, and the maximum of the correlations between the permuted trait and the expression of the genes was computed. This was done a thousand times; the thousand maxima so obtained formed the empirical null distribution against which the significance of a correlation was tested.

### Subjects for locomotion in open field test

Six-week-old male mice (*n*>10 per strain, see section “animals, slice preparation and recording” for strain names) arrived in the facility in different batches in a period spanning 2 years. Mice were housed individually in Macrolon cages on sawdust bedding, which were, for the purpose of animal welfare, enriched with cardboard nesting material and a curved PVC tube. Food (Harlan Teklad) and water was provided ad libitum. All mice were habituated to the facility for at least 7 days before testing started. Prior to the open field testing described below, mice had been exposed to novelty tests in the home cage, an elevated plus maze and a light dark box apparatus, as described previously [79]. Housing and testing rooms were controlled for temperature, humidity and light-dark cycle (7 AM lights on, 7 PM lights off; testing during the light phase).

### Locomotion in open field

All experimental procedures were approved by the local animal research committee and complied with the European Council Directive (86/609/EEC). Mice were introduced into a corner of a white square open field (50×50 cm, walls 35 cm high) illuminated with a single white fluorescent light bulb from above (130 lx), and exploration was tracked for 10 minutes (12.5 frames/s; EthoVision 3.0, Noldus Information Technology). The SEE software (Strategy for the Exploration of Exploration [42], [80] was used to smoothen path shape to calculate the total distance moved. Furthermore, SEE uses the distribution of speed peaks to parse the locomotor data into lingering segments (slow local movements) and progression segments, which together constitute the total distance moved.

## Supporting Information

Figure S1 **Zoom in of the QTL for the trait Amplitude 1–45 Hz (ACSF), located at Chr4 40.937–49.610 Mb.** The LRS scores (*y*-axis) quantify the relation between genomic markers (*x*-axis) and the trait. Parental allele effect is shown in green and red: a green line indicates that DBA/2J alleles increase trait values. A red line indicates that C57BL/6J alleles increase trait values.
(PNG)

Figure S2 **Zoom in of the QTL for the trait Amplitude 1–45 Hz (ACSF), located at Chr4 53.915–65.605 Mb.** The LRS scores (*y*-axis) quantify the relation between genomic markers (*x*-axis) and the trait. Parental allele effect is shown in green and red: a green line indicates that DBA/2J alleles increase trait values. A red line indicates that C57BL/6J alleles increase trait values.
(PNG)

Figure S3 **Zoom in of the QTL for the trait Amplitude 1–45 Hz (ACSF), located at Chr5 16.516–22.717 Mb.** The LRS scores (*y*-axis) quantify the relation between genomic markers (*x*-axis) and the trait. Parental allele effect is shown in green and red: a green line indicates that DBA/2J alleles increase trait values. A red line indicates that C57BL/6J alleles increase trait values.
(PNG)

Figure S4 **Zoom in of the QTL for the trait Correlation (ACSF), located at Chr4 38.926–44.246 Mb.** The LRS scores (*y*-axis) quantify the relation between genomic markers (*x*-axis) and the trait. Parental allele effect is shown in green and red: a green line indicates that DBA/2J alleles increase trait values. A red line indicates that C57BL/6J alleles increase trait values.
(PNG)

Figure S5 **Zoom in of the QTL for the trait Correlation (ACSF), located at Chr4 58.377–62.347 Mb.** The LRS scores (*y*-axis) quantify the relation between genomic markers (*x*-axis) and the trait. Parental allele effect is shown in green and red: a green line indicates that DBA/2J alleles increase trait values. A red line indicates that C57BL/6J alleles increase trait values.
(PNG)

Figure S6 **Zoom in of the QTL for the trait Correlation (ACSF), located at Chr14 56.052–59.824 Mb.** The LRS scores (*y*-axis) quantify the relation between genomic markers (*x*-axis) and the trait. Parental allele effect is shown in green and red: a green line indicates that DBA/2J alleles increase trait values. A red line indicates that C57BL/6J alleles increase trait values.
(PNG)

Figure S7 **Zoom in of the QTL for the trait Amplitude 1–45 Hz (CCH), located at Chr1 156.053–160.478 Mb.** The LRS scores (*y*-axis) quantify the relation between genomic markers (*x*-axis) and the trait. Parental allele effect is shown in green and red: a green line indicates that DBA/2J alleles increase trait values. A red line indicates that C57BL/6J alleles increase trait values.
(PNG)

Figure S8 **Zoom in of the QTL for the trait Amplitude 1–45 Hz (CCH), located at Chr1 109.358–128.626 Mb.** The LRS scores (*y*-axis) quantify the relation between genomic markers (*x*-axis) and the trait. Parental allele effect is shown in green and red: a green line indicates that DBA/2J alleles increase trait values. A red line indicates that C57BL/6J alleles increase trait values.
(PNG)

Figure S9 **Zoom in of the QTL for the trait Amplitude 1–45 Hz (CCH), located at Chr2 57.639–60.486 Mb.** The LRS scores (*y*-axis) quantify the relation between genomic markers (*x*-axis) and the trait. Parental allele effect is shown in green and red: a green line indicates that DBA/2J alleles increase trait values. A red line indicates that C57BL/6J alleles increase trait values.
(PNG)

Figure S10 **Zoom in of the QTL for the trait Amplitude 1–45 Hz (CCH), located at Chr2 65.6704–72.240 Mb.** The LRS scores (*y*-axis) quantify the relation between genomic markers (*x*-axis) and the trait. Parental allele effect is shown in green and red: a green line indicates that DBA/2J alleles increase trait values. A red line indicates that C57BL/6J alleles increase trait values.
(PNG)

Figure S11 **Zoom in of the QTL for the trait Amplitude 1–45 Hz (CCH), located at Chr5 3.143–12.371 Mb.** The LRS scores (*y*-axis) quantify the relation between genomic markers (*x*-axis) and the trait. Parental allele effect is shown in green and red: a green line indicates that DBA/2J alleles increase trait values. A red line indicates that C57BL/6J alleles increase trait values.
(PNG)

Figure S12 **Zoom in of the QTL for the trait Peak amplitude, located at Chr2 19.000–25.727 Mb.** The LRS scores (*y*-axis) quantify the relation between genomic markers (*x*-axis) and the trait. Parental allele effect is shown in green and red: a green line indicates that DBA/2J alleles increase trait values. A red line indicates that C57BL/6J alleles increase trait values.
(PNG)

Figure S13 **Zoom in of the QTL for the trait Peak amplitude, located at Chr2 132.641–135.954 MB.** The LRS scores (*y*-axis) quantify the relation between genomic markers (*x*-axis) and the trait. Parental allele effect is shown in green and red: a green line indicates that DBA/2J alleles increase trait values. A red line indicates that C57BL/6J alleles increase trait values.
(PNG)

Figure S14 **Zoom in of the QTL for the trait Peak amplitude, located at Chr5 3.143–20.086 Mb.** The LRS scores (*y*-axis) quantify the relation between genomic markers (*x*-axis) and the trait. Parental allele effect is shown in green and red: a green line indicates that DBA/2J alleles increase trait values. A red line indicates that C57BL/6J alleles increase trait values.
(PNG)

Figure S15 **Zoom in of the QTL for the trait Correlation (CCH), located at Chr2 76.832–80.436 Mb.** The LRS scores (*y*-axis) quantify the relation between genomic markers (*x*-axis) and the trait. Parental allele effect is shown in green and red: a green line indicates that DBA/2J alleles increase trait values. A red line indicates that C57BL/6J alleles increase trait values.
(PNG)

Figure S16 **Zoom in of the QTL for the trait Correlation (CCH), located at Chr2 133.463–135.918 Mb.** The LRS scores (*y*-axis) quantify the relation between genomic markers (*x*-axis) and the trait. Parental allele effect is shown in green and red: a green line indicates that DBA/2J alleles increase trait values. A red line indicates that C57BL/6J alleles increase trait values.
(PNG)

Figure S17 **Zoom in of the QTL for the trait Correlation (CCH), located at Chr5, Chr5 4.468–12.371 Mb.** The LRS scores (*y*-axis) quantify the relation between genomic markers (*x*-axis) and the trait. Parental allele effect is shown in green and red: a green line indicates that DBA/2J alleles increase trait values. A red line indicates that C57BL/6J alleles increase trait values.
(PNG)

Figure S18 **Zoom in of the QTL for the trait Peak frequency, located at Chr12 30.140–35.762 Mb.** The LRS scores (*y*-axis) quantify the relation between genomic markers (*x*-axis) and the trait. Parental allele effect is shown in green and red: a green line indicates that DBA/2J alleles increase trait values. A red line indicates that C57BL/6J alleles increase trait values.
(PNG)

Figure S19 **Zoom in of the QTL for the trait Peak frequency, located Chr17 72.196–76.447 Mb.** The LRS scores (*y*-axis) quantify the relation between genomic markers (*x*-axis) and the trait. Parental allele effect is shown in green and red: a green line indicates that DBA/2J alleles increase trait values. A red line indicates that C57BL/6J alleles increase trait values.
(PNG)

Table S1 **Heritability scores (** ***h*** **) and** ***P-*** **values from** ***F*** **statistics from the ANOVAs of all the traits derived in the ACSF condition (spontaneous activity).** The trait names are coded: Amplitude a_b_Hz_c indicates the integrated amplitude between a and b Hz, in region number c; Corr(a,b) indicates the correlation of activity between region a and region b. The numbers refer to the following regions: 1 = CA3 stratum radiatum/lacunosum moleculare, 2 = CA3 stratum pyramidale, 3 = CA3 stratum oriens, 4 = CA1 stratum radiatum/lacunosum moleculare, 5 = CA1 stratum pyramidale, 6 = CA1 stratum oriens, 7 = Dentate Gyrus hilus, 8 = Dentate Gyrus stratum granulosum, 9 = Dentate Gyrus stratum moleculare.
(XLS)

Table S2 **Heritability scores (** ***h*** **) and** ***P-*** **values from** ***F*** **statistics from the ANOVAs of all the traits derived in the carbachol condition (oscillations).** The trait names are coded: Amplitude a_b_Hz_c indicates the integrated amplitude between a and b Hz, in region c. Amplitude_a is the peak amplitude in region a, Frequency_a indicates the peak frequency in region a. PLF(a,b) is the phase locking factor of the activity between region a and region b. The numbers refer to the following regions: 1 = CA3 stratum radiatum/lacunosum moleculare, 2 = CA3 stratum pyramidale, 3 = CA3 stratum oriens, 4 = CA1 stratum radiatum/lacunosum moleculare, 5 = CA1 stratum pyramidale, 6 = CA1 stratum oriens, 7 = Dentate Gyrus hilus, 8 = Dentate Gyrus stratum granulosum, 9 = Dentate Gyrus stratum moleculare.
(XLS)

Table S3 **Description and IDs of first subset of phenotypes from the GeneNetwork phenotype database: physiological hippocampal traits.**
(XLS)

Table S4 **Description and IDs of second subset of phenotypes from the GeneNetwork phenotype database: behavioral traits.**
(XLS)

Table S5 **List of genes in a QTL for the trait Amplitude 1–45 Hz (ACSF), located at Chr4 40.937–49.610 Mb.**
(XLS)

Table S6 **List of genes in a QTL for the trait Amplitude 1–45 Hz (ACSF), located at Chr4 53.915–65.605 Mb.**
(XLS)

Table S7 **List of genes in a QTL for the trait Amplitude 1–45 Hz (ACSF), located at Chr5 16.516–22.717 Mb.**
(XLS)

Table S8 **List of genes in a QTL for the trait Correlation (ACSF), located at Chr4 38.926–44.246 Mb.**
(XLS)

Table S9 **List of genes in a QTL for the trait Correlation (ACSF), located at Chr4 58.377–62.347 Mb.**
(XLS)

Table S10 **List of genes in a QTL for the trait Correlation (ACSF), located at Chr14 56.052–59.824 Mb.**
(XLS)

Table S11 **List of genes in a QTL for the trait Amplitude 1–45 Hz (CCH), located at Chr1 156.053–160.478 Mb.**
(XLS)

Table S12 **List of genes in a QTL for the trait Amplitude 1–45 Hz (CCH), located at Chr1 109.358–128.626 Mb.**
(XLS)

Table S13 **List of genes in a QTL for the trait Amplitude 1–45 Hz (CCH), located at Chr2 57.639–60.486 Mb.**
(XLS)

Table S14 **List of genes in a QTL for the trait Amplitude 1–45 Hz (CCH), located at Chr2 65.6704–72.240 Mb.**
(XLS)

Table S15 **List of genes in a QTL for the trait Amplitude 1–45 Hz (CCH), located at Chr5 3.143–12.371 Mb.**
(XLS)

Table S16 **List of genes in a QTL for the trait Peak amplitude, located at Chr2 19.000–25.727 Mb.**
(XLS)

Table S17 **List of genes in a QTL for the trait Peak amplitude, located at Chr2 132.641–135.954 MB**
(XLS)

Table S18 **List of genes in a QTL for the trait Peak amplitude, located at Chr5 3.143–20.086 Mb.**
(XLS)

Table S19 **List of genes in a QTL for the trait Correlation (CCH), located at Chr2 76.832–80.436 Mb.**
(XLS)

Table S20 **List of genes in a QTL for the trait Correlation (CCH), located at Chr2 133.463–135.918 Mb.**
(XLS)

Table S21 **List of genes in a QTL for the trait Correlation (CCH), located at Chr5, Chr5 4.468–12.371 Mb.**
(XLS)

Table S22 **List of genes in a QTL for the trait Peak frequency, located Chr17 72.196–76.447 Mb.**
(XLS)

Table S23 **List of genes in a QTL for the trait Peak frequency, located at Chr12 30.140–35.762 Mb.**
(XLS)

Table S24 **The locations of the 19 QTLs.**
(XLS)
