# Supplementary figures and images for: Novel Candidate Genes Associated with Hippocampal Oscillations
Source: PLoS One. 2011 Oct 31;6(10):e26586. doi: 10.1371/journal.pone.0026586 (PMC3204991; doi:10.1371/journal.pone.0026586)

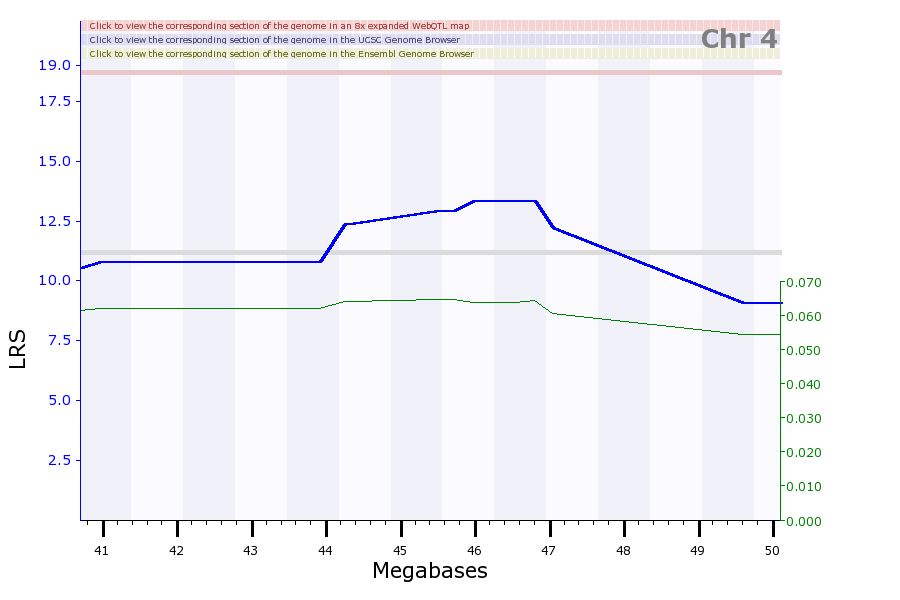

Supplement: Figure S1 — Zoom in of the QTL for the trait Amplitude 1–45 Hz (ACSF), located at Chr4 40.937–49.610 Mb. The LRS scores (y-axis) quantify the relation between genomic markers (x-axis) and the trait. Parental allele effect is shown in green and red: a green line indicates that DBA/2J alleles increase trait values. A red line indicates that C57BL/6J alleles increase trait values. (PNG) [file pone.0026586.s001.png]

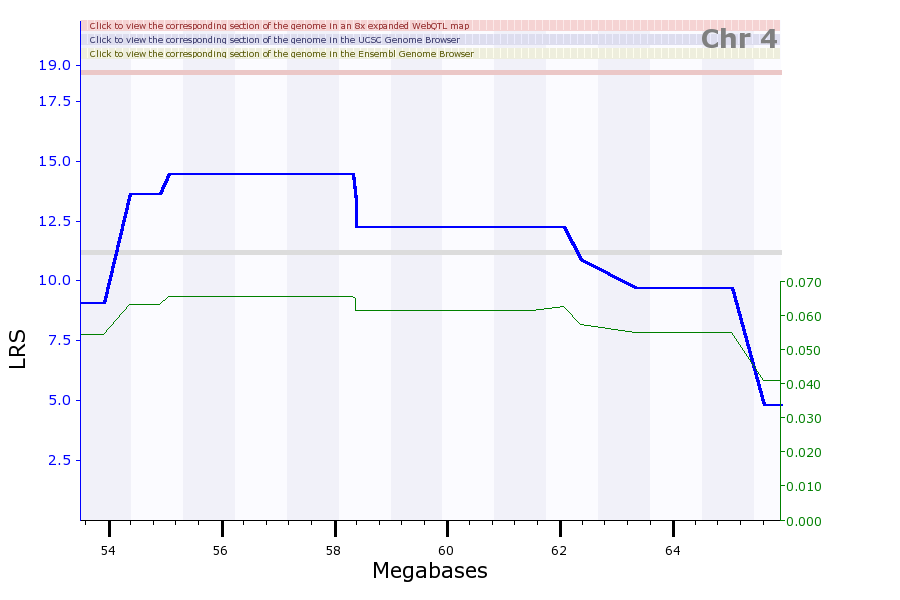

Supplement: Figure S2 — Zoom in of the QTL for the trait Amplitude 1–45 Hz (ACSF), located at Chr4 53.915–65.605 Mb. The LRS scores (y-axis) quantify the relation between genomic markers (x-axis) and the trait. Parental allele effect is shown in green and red: a green line indicates that DBA/2J alleles increase trait values. A red line indicates that C57BL/6J alleles increase trait values. (PNG) [file pone.0026586.s002.png]

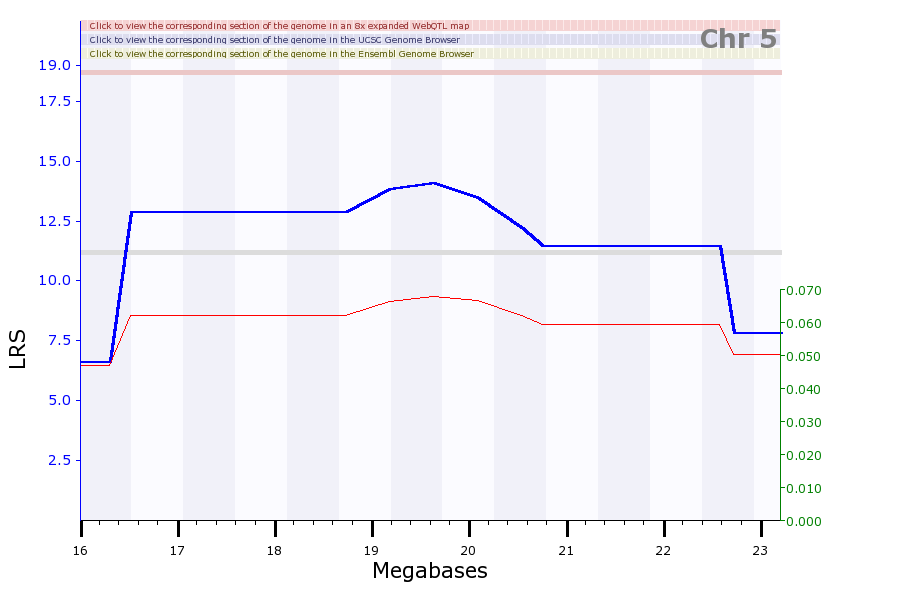

Supplement: Figure S3 — Zoom in of the QTL for the trait Amplitude 1–45 Hz (ACSF), located at Chr5 16.516–22.717 Mb. The LRS scores (y-axis) quantify the relation between genomic markers (x-axis) and the trait. Parental allele effect is shown in green and red: a green line indicates that DBA/2J alleles increase trait values. A red line indicates that C57BL/6J alleles increase trait values. (PNG) [file pone.0026586.s003.png]

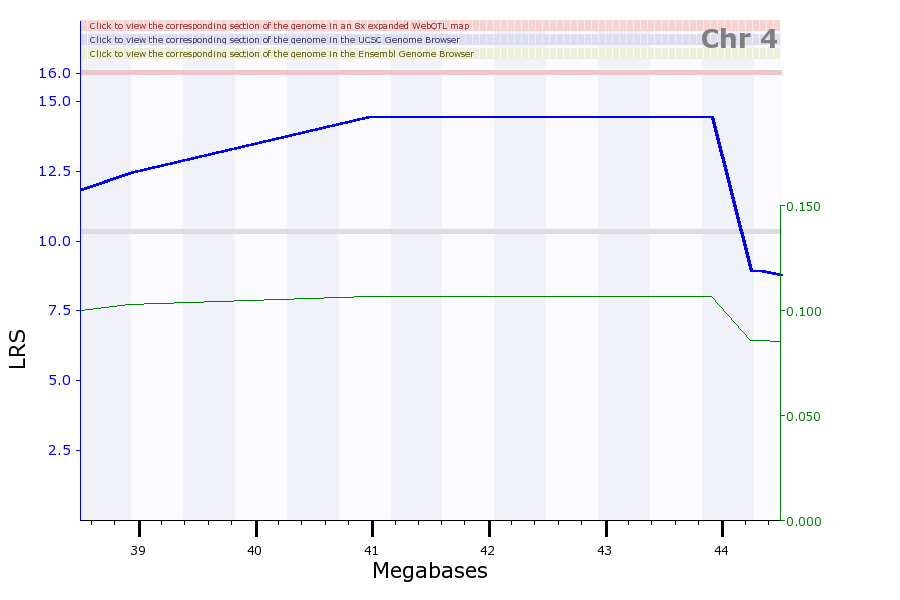

Supplement: Figure S4 — Zoom in of the QTL for the trait Correlation (ACSF), located at Chr4 38.926–44.246 Mb. The LRS scores (y-axis) quantify the relation between genomic markers (x-axis) and the trait. Parental allele effect is shown in green and red: a green line indicates that DBA/2J alleles increase trait values. A red line indicates that C57BL/6J alleles increase trait values. (PNG) [file pone.0026586.s004.png]

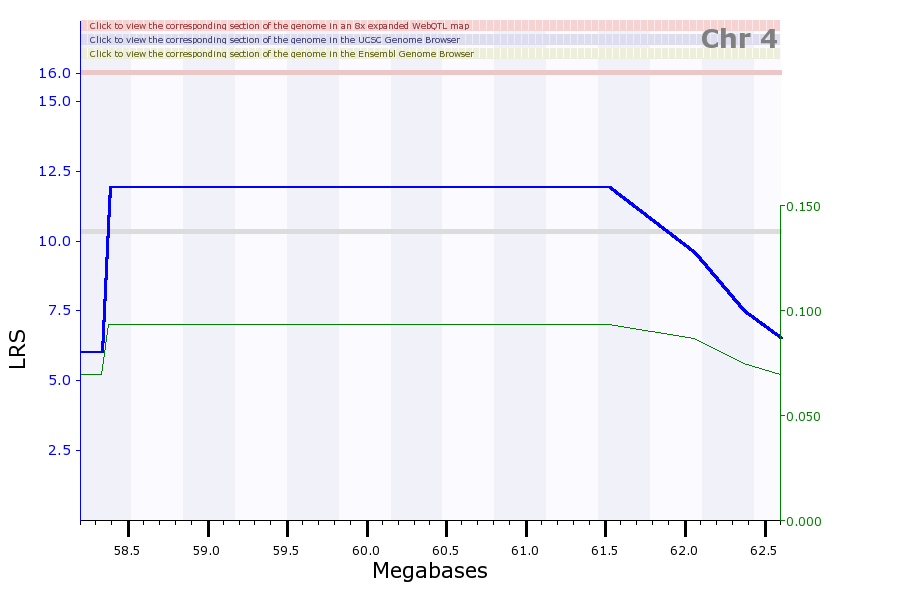

Supplement: Figure S5 — Zoom in of the QTL for the trait Correlation (ACSF), located at Chr4 58.377–62.347 Mb. The LRS scores (y-axis) quantify the relation between genomic markers (x-axis) and the trait. Parental allele effect is shown in green and red: a green line indicates that DBA/2J alleles increase trait values. A red line indicates that C57BL/6J alleles increase trait values. (PNG) [file pone.0026586.s005.png]

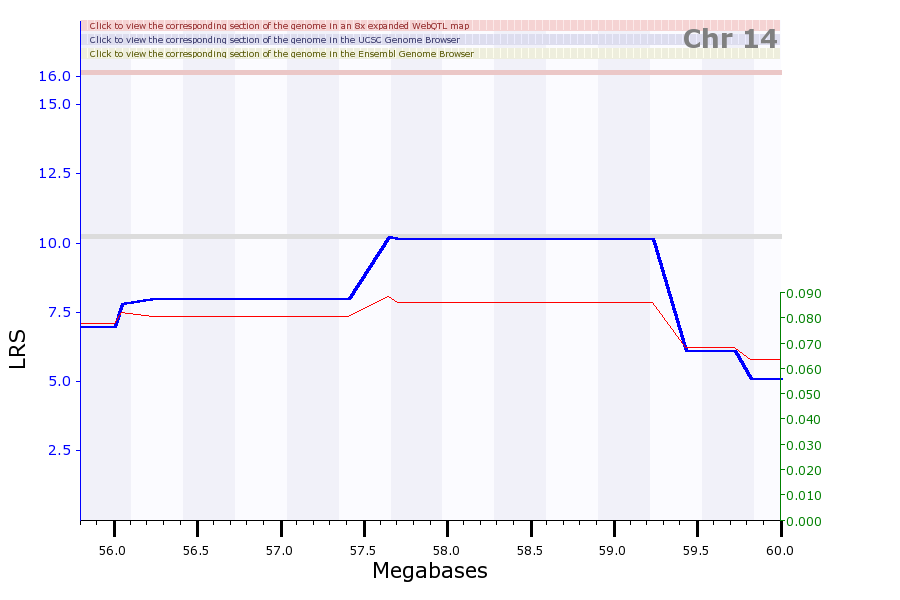

Supplement: Figure S6 — Zoom in of the QTL for the trait Correlation (ACSF), located at Chr14 56.052–59.824 Mb. The LRS scores (y-axis) quantify the relation between genomic markers (x-axis) and the trait. Parental allele effect is shown in green and red: a green line indicates that DBA/2J alleles increase trait values. A red line indicates that C57BL/6J alleles increase trait values. (PNG) [file pone.0026586.s006.png]

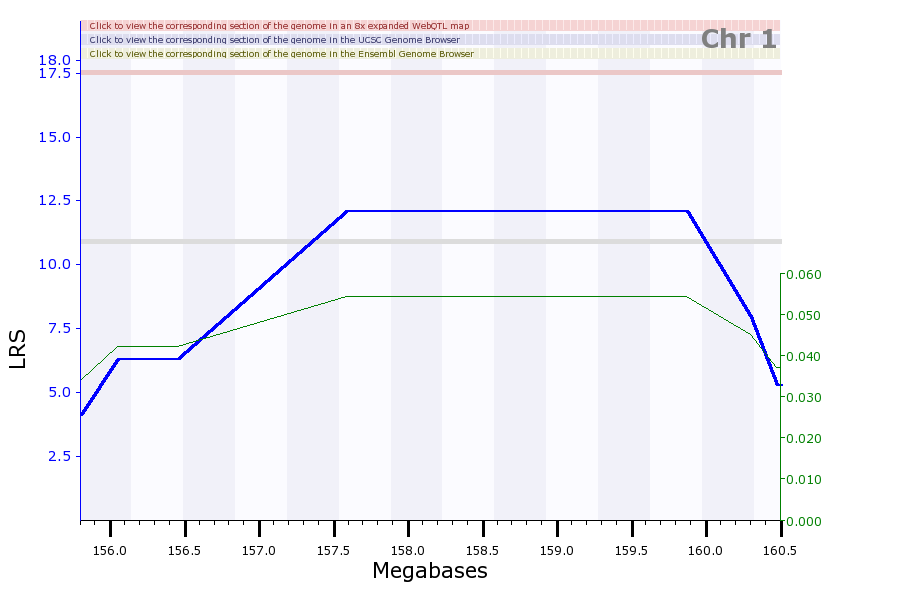

Supplement: Figure S7 — Zoom in of the QTL for the trait Amplitude 1–45 Hz (CCH), located at Chr1 156.053–160.478 Mb. The LRS scores (y-axis) quantify the relation between genomic markers (x-axis) and the trait. Parental allele effect is shown in green and red: a green line indicates that DBA/2J alleles increase trait values. A red line indicates that C57BL/6J alleles increase trait values. (PNG) [file pone.0026586.s007.png]

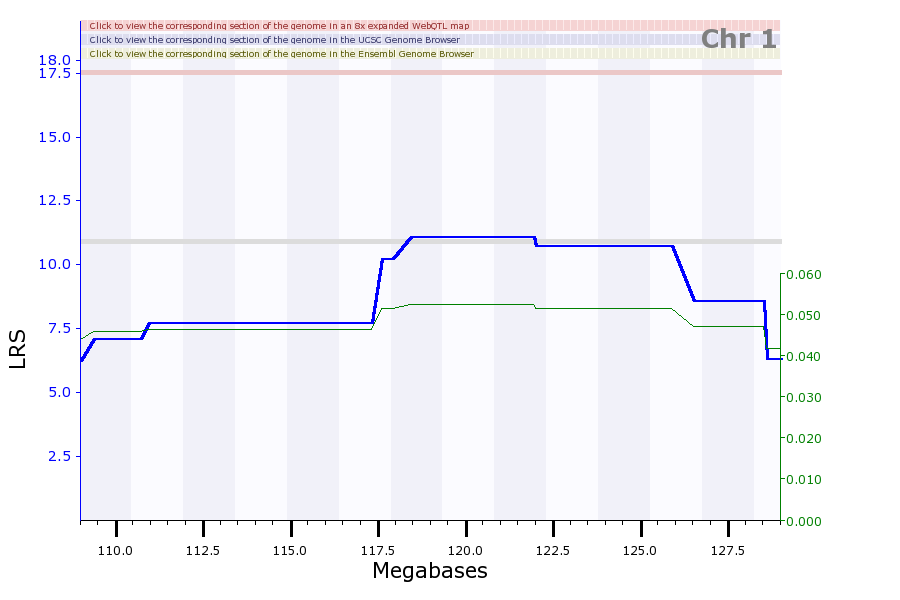

Supplement: Figure S8 — Zoom in of the QTL for the trait Amplitude 1–45 Hz (CCH), located at Chr1 109.358–128.626 Mb. The LRS scores (y-axis) quantify the relation between genomic markers (x-axis) and the trait. Parental allele effect is shown in green and red: a green line indicates that DBA/2J alleles increase trait values. A red line indicates that C57BL/6J alleles increase trait values. (PNG) [file pone.0026586.s008.png]

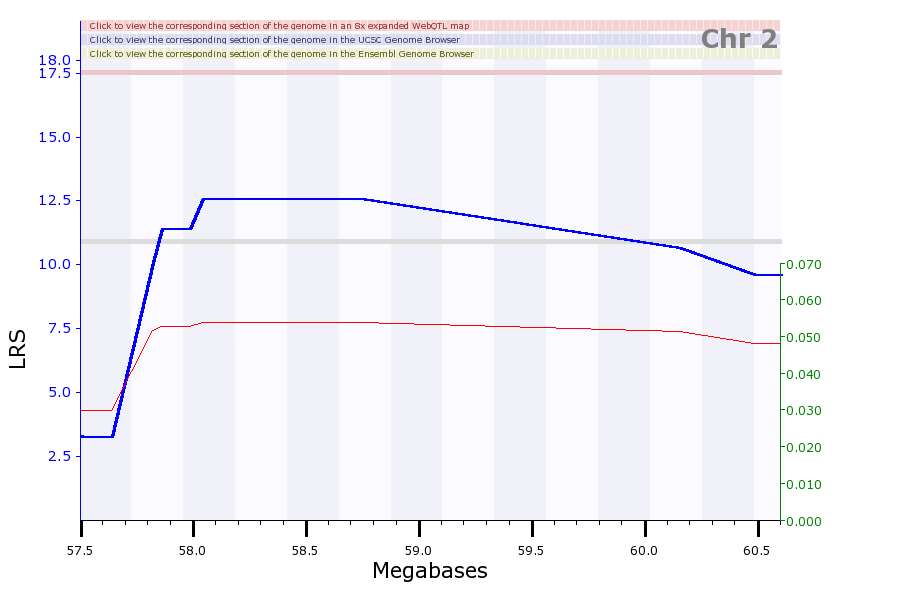

Supplement: Figure S9 — Zoom in of the QTL for the trait Amplitude 1–45 Hz (CCH), located at Chr2 57.639–60.486 Mb. The LRS scores (y-axis) quantify the relation between genomic markers (x-axis) and the trait. Parental allele effect is shown in green and red: a green line indicates that DBA/2J alleles increase trait values. A red line indicates that C57BL/6J alleles increase trait values. (PNG) [file pone.0026586.s009.png]

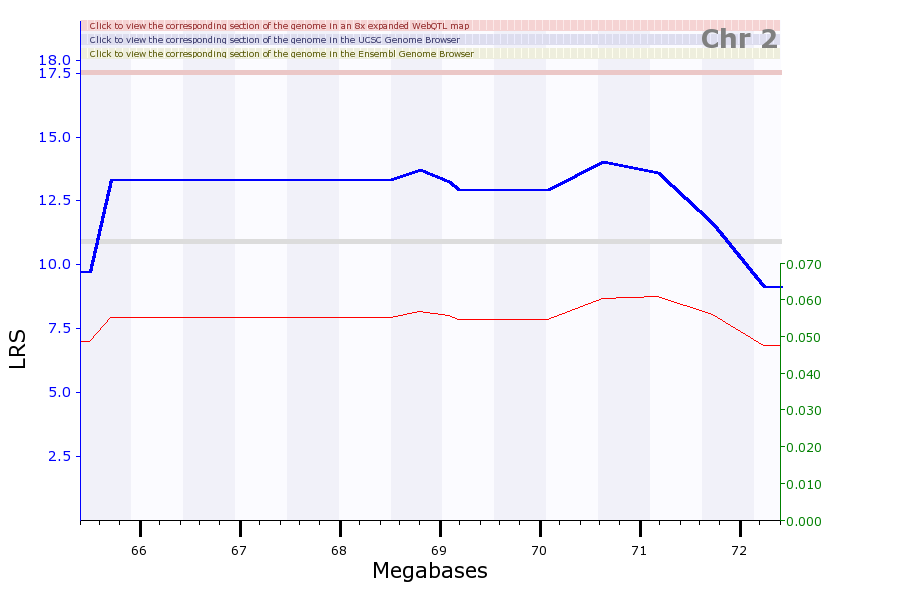

Supplement: Figure S10 — Zoom in of the QTL for the trait Amplitude 1–45 Hz (CCH), located at Chr2 65.6704–72.240 Mb. The LRS scores (y-axis) quantify the relation between genomic markers (x-axis) and the trait. Parental allele effect is shown in green and red: a green line indicates that DBA/2J alleles increase trait values. A red line indicates that C57BL/6J alleles increase trait values. (PNG) [file pone.0026586.s010.png]

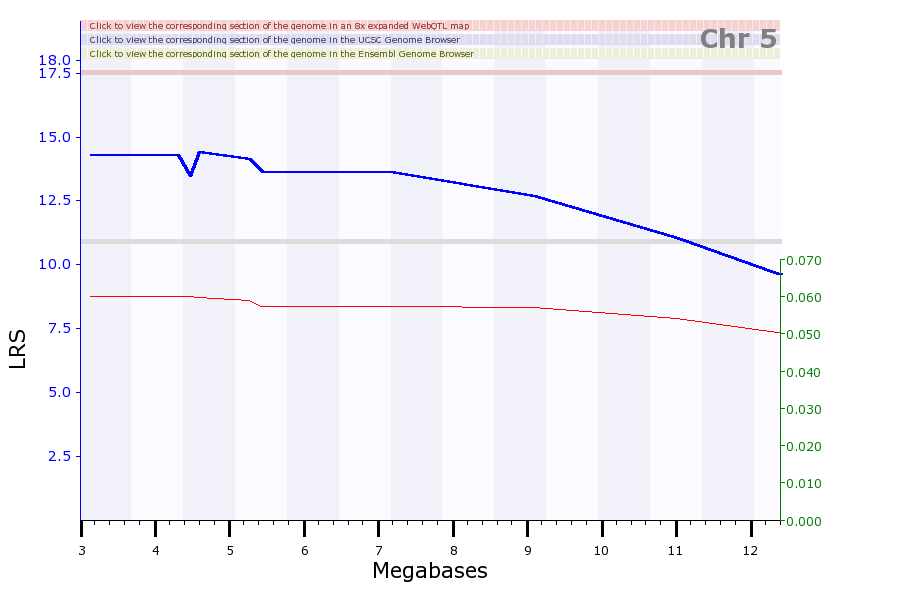

Supplement: Figure S11 — Zoom in of the QTL for the trait Amplitude 1–45 Hz (CCH), located at Chr5 3.143–12.371 Mb. The LRS scores (y-axis) quantify the relation between genomic markers (x-axis) and the trait. Parental allele effect is shown in green and red: a green line indicates that DBA/2J alleles increase trait values. A red line indicates that C57BL/6J alleles increase trait values. (PNG) [file pone.0026586.s011.png]

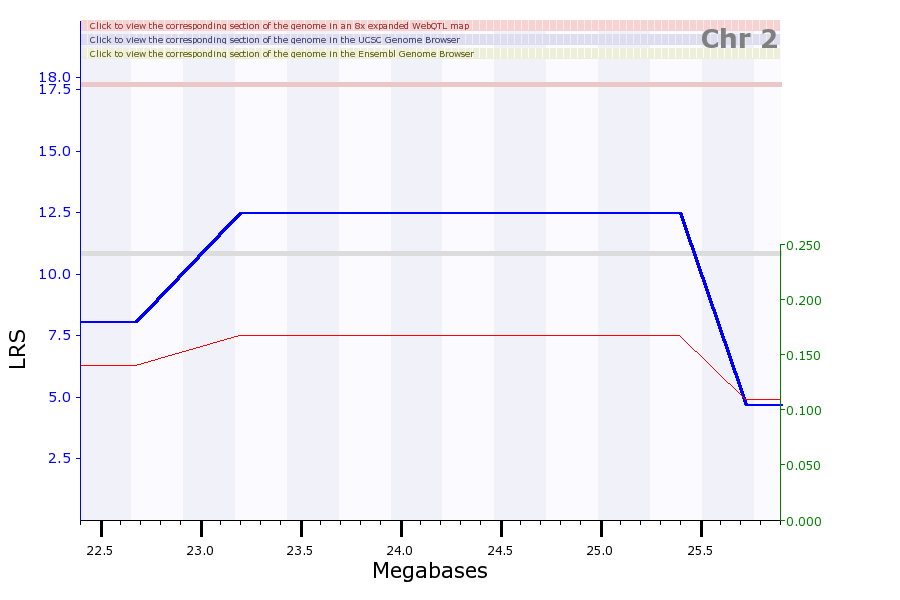

Supplement: Figure S12 — Zoom in of the QTL for the trait Peak amplitude, located at Chr2 19.000–25.727 Mb. The LRS scores (y-axis) quantify the relation between genomic markers (x-axis) and the trait. Parental allele effect is shown in green and red: a green line indicates that DBA/2J alleles increase trait values. A red line indicates that C57BL/6J alleles increase trait values. (PNG) [file pone.0026586.s012.png]

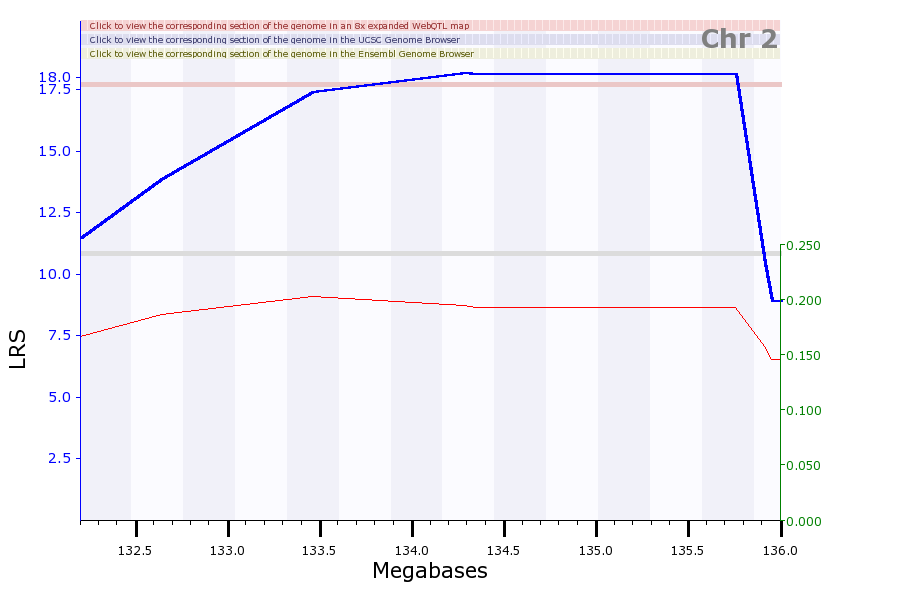

Supplement: Figure S13 — Zoom in of the QTL for the trait Peak amplitude, located at Chr2 132.641–135.954 MB. The LRS scores (y-axis) quantify the relation between genomic markers (x-axis) and the trait. Parental allele effect is shown in green and red: a green line indicates that DBA/2J alleles increase trait values. A red line indicates that C57BL/6J alleles increase trait values. (PNG) [file pone.0026586.s013.png]

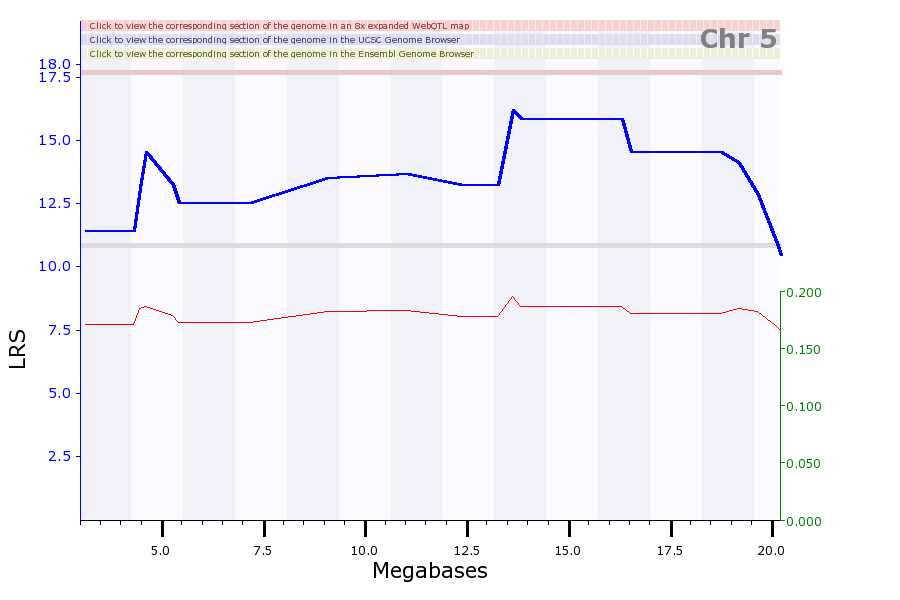

Supplement: Figure S14 — Zoom in of the QTL for the trait Peak amplitude, located at Chr5 3.143–20.086 Mb. The LRS scores (y-axis) quantify the relation between genomic markers (x-axis) and the trait. Parental allele effect is shown in green and red: a green line indicates that DBA/2J alleles increase trait values. A red line indicates that C57BL/6J alleles increase trait values. (PNG) [file pone.0026586.s014.png]

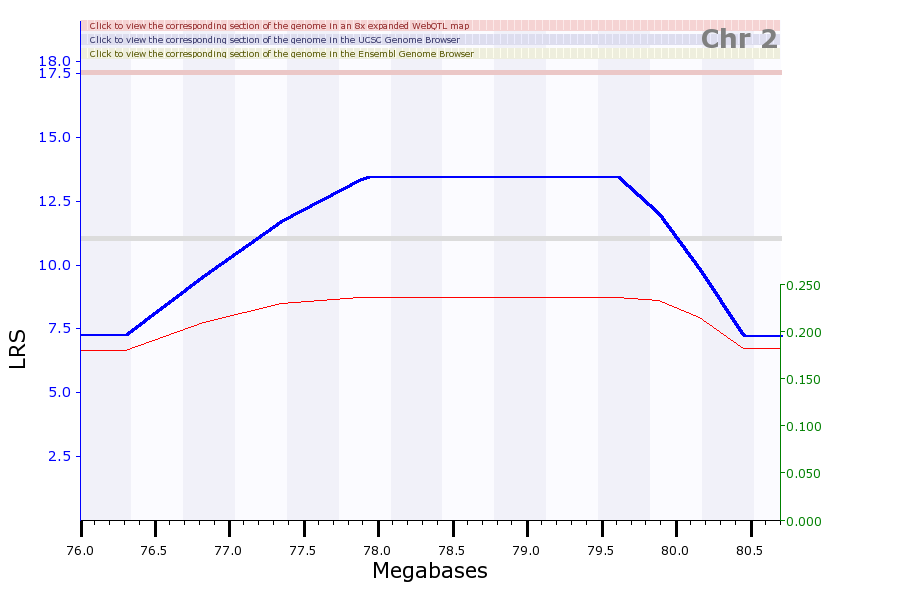

Supplement: Figure S15 — Zoom in of the QTL for the trait Correlation (CCH), located at Chr2 76.832–80.436 Mb. The LRS scores (y-axis) quantify the relation between genomic markers (x-axis) and the trait. Parental allele effect is shown in green and red: a green line indicates that DBA/2J alleles increase trait values. A red line indicates that C57BL/6J alleles increase trait values. (PNG) [file pone.0026586.s015.png]

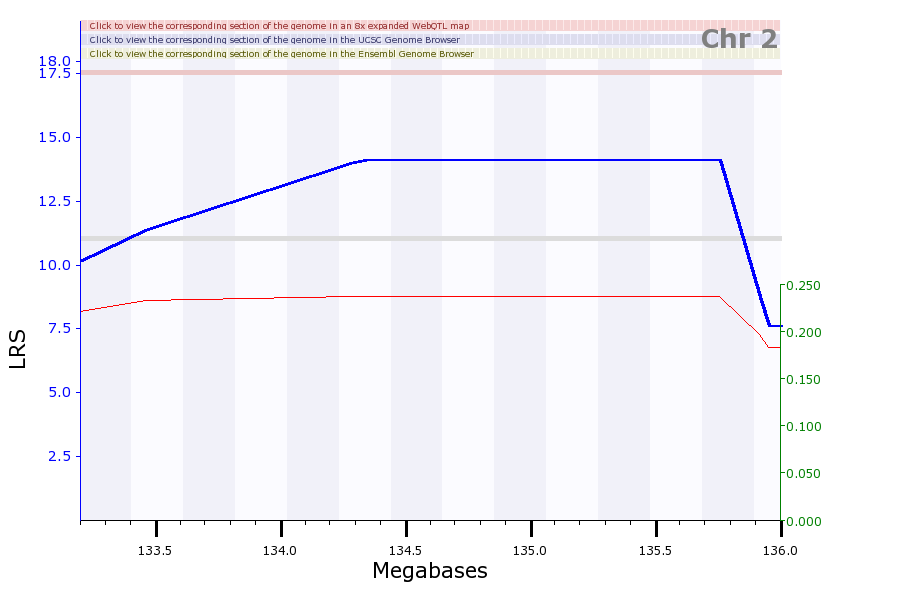

Supplement: Figure S16 — Zoom in of the QTL for the trait Correlation (CCH), located at Chr2 133.463–135.918 Mb. The LRS scores (y-axis) quantify the relation between genomic markers (x-axis) and the trait. Parental allele effect is shown in green and red: a green line indicates that DBA/2J alleles increase trait values. A red line indicates that C57BL/6J alleles increase trait values. (PNG) [file pone.0026586.s016.png]

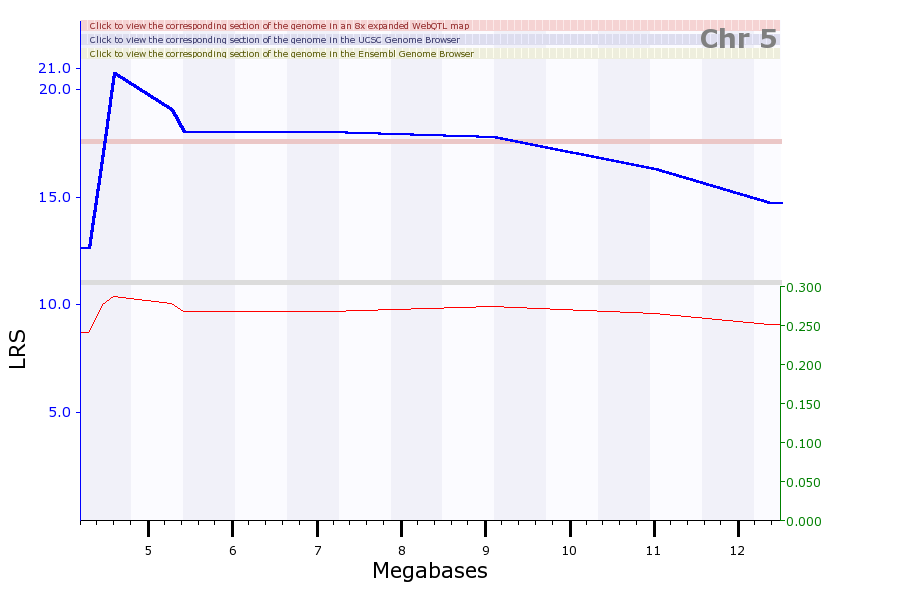

Supplement: Figure S17 — Zoom in of the QTL for the trait Correlation (CCH), located at Chr5, Chr5 4.468–12.371 Mb. The LRS scores (y-axis) quantify the relation between genomic markers (x-axis) and the trait. Parental allele effect is shown in green and red: a green line indicates that DBA/2J alleles increase trait values. A red line indicates that C57BL/6J alleles increase trait values. (PNG) [file pone.0026586.s017.png]

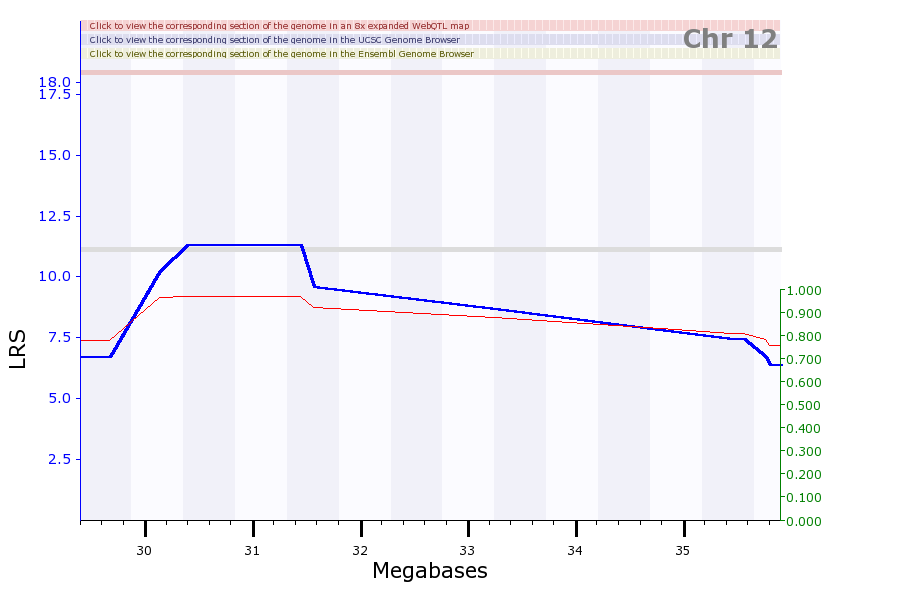

Supplement: Figure S18 — Zoom in of the QTL for the trait Peak frequency, located at Chr12 30.140–35.762 Mb. The LRS scores (y-axis) quantify the relation between genomic markers (x-axis) and the trait. Parental allele effect is shown in green and red: a green line indicates that DBA/2J alleles increase trait values. A red line indicates that C57BL/6J alleles increase trait values. (PNG) [file pone.0026586.s018.png]

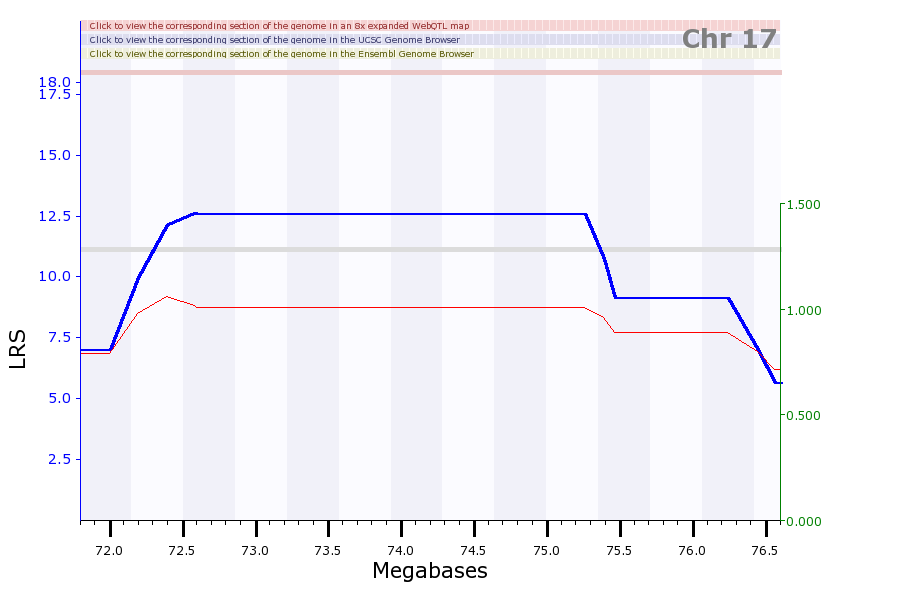

Supplement: Figure S19 — Zoom in of the QTL for the trait Peak frequency, located Chr17 72.196–76.447 Mb. The LRS scores (y-axis) quantify the relation between genomic markers (x-axis) and the trait. Parental allele effect is shown in green and red: a green line indicates that DBA/2J alleles increase trait values. A red line indicates that C57BL/6J alleles increase trait values. (PNG) [file pone.0026586.s019.png]
