# Supplementary material for: Novel Candidate Genes Associated with Hippocampal Oscillations
Source: PLoS One. 2011 Oct 31;6(10):e26586. doi: 10.1371/journal.pone.0026586 (PMC3204991; doi:10.1371/journal.pone.0026586)
Supplement: Table S1 — Heritability scores ( h ) and P- values from F statistics from the ANOVAs of all the traits derived in the ACSF condition (spontaneous activity). The trait names are coded: Amplitude a_b_Hz_c indicates the integrated amplitude between a and b Hz, in region number c; Corr(a,b) indicates the correlation of activity between region a and region b. The numbers refer to the following regions: 1 = CA3 stratum radiatum/lacunosum moleculare, 2 = CA3 stratum pyramidale, 3 = CA3 stratum oriens, 4 = CA1 stratum radiatum/lacunosum moleculare, 5 = CA1 stratum pyramidale, 6 = CA1 stratum oriens, 7 = Dentate Gyrus hilus, 8 = Dentate Gyrus stratum granulosum, 9 = Dentate Gyrus stratum moleculare. (XLS) [file pone.0026586.s020.xls]

trait	ANOVA	*h*		trait	ANOVA	*h*		trait	ANOVA	*h*
1_4_Hz_1	p < 1e-10	0,07		1_4_Hz_6	p < 1e-10	0,09		corr(1,8)	p < 1e-9	0,07
4_7_Hz_1	p < 1e-10	0,08		4_7_Hz_6	p < 1e-10	0,19		corr(1,9)	p < 1e-9	0,06
7_13_Hz_1	p < 1e-10	0,09		7_13_Hz_6	p < 1e-10	0,23		corr(2,3)	p < 1e-10	0,08
13_25_Hz_1	p < 1e-10	0,12		13_25_Hz_6	p < 1e-10	0,27		corr(2,4)	p < 1e-10	0,07
25_35_Hz_1	p < 1e-10	0,16		25_35_Hz_6	p < 1e-10	0,26		corr(2,5)	p < 1e-10	0,11
35_45_Hz_1	p < 1e-10	0,20		35_45_Hz_6	p < 1e-10	0,27		corr(2,6)	p < 1e-10	0,11
1_4_Hz_2	p < 1e-9	0,06		1_4_Hz_7	p < 1e-10	0,09		corr(2,7)	p < 1e-10	0,11
4_7_Hz_2	p < 1e-10	0,11		4_7_Hz_7	p < 1e-10	0,15		corr(2,8)	p < 1e-10	0,12
7_13_Hz_2	p < 1e-10	0,16		7_13_Hz_7	p < 1e-10	0,18		corr(2,9)	p < 1e-10	0,11
13_25_Hz_2	p < 1e-10	0,18		13_25_Hz_7	p < 1e-10	0,22		corr(3,4)	p < 1e-8	0,06
25_35_Hz_2	p < 1e-10	0,17		25_35_Hz_7	p < 1e-10	0,24		corr(3,5)	p < 1e-10	0,10
35_45_Hz_2	p < 1e-10	0,18		35_45_Hz_7	p < 1e-10	0,25		corr(3,6)	p < 1e-10	0,10
1_4_Hz_3	p < 1e-10	0,10		1_4_Hz_8	p < 1e-10	0,08		corr(3,7)	p < 1e-10	0,11
4_7_Hz_3	p < 1e-10	0,18		4_7_Hz_8	p < 1e-10	0,17		corr(3,8)	p < 1e-10	0,12
7_13_Hz_3	p < 1e-10	0,24		7_13_Hz_8	p < 1e-10	0,20		corr(3,9)	p < 1e-10	0,12
13_25_Hz_3	p < 1e-10	0,27		13_25_Hz_8	p < 1e-10	0,23		corr(4,5)	p < 1e-8	0,06
25_35_Hz_3	p < 1e-10	0,28		25_35_Hz_8	p < 1e-10	0,25		corr(4,6)	p < 1e-10	0,08
35_45_Hz_3	p < 1e-10	0,27		35_45_Hz_8	p < 1e-10	0,25		corr(4,7)	p < 1e-9	0,06
1_4_Hz_4	p < 1e-10	0,07		1_4_Hz_9	p < 1e-10	0,08		corr(4,8)	p < 1e-9	0,06
4_7_Hz_4	p < 1e-10	0,12		4_7_Hz_9	p < 1e-10	0,15		corr(4,9)	p < 1e-10	0,08
7_13_Hz_4	p < 1e-10	0,14		7_13_Hz_9	p < 1e-10	0,21		corr(5,6)	p < 1e-10	0,10
13_25_Hz_4	p < 1e-10	0,17		13_25_Hz_9	p < 1e-10	0,25		corr(5,7)	p < 1e-10	0,10
25_35_Hz_4	p < 1e-10	0,21		25_35_Hz_9	p < 1e-10	0,26		corr(5,8)	p < 1e-10	0,09
35_45_Hz_4	p < 1e-10	0,24		35_45_Hz_9	p < 1e-10	0,26		corr(5,9)	p < 1e-10	0,11
1_4_Hz_5	p < 1e-10	0,09		corr(1,2)	p < 1e-9	0,06		corr(6,7)	p < 1e-10	0,11
4_7_Hz_5	p < 1e-10	0,16		corr(1,3)	p < 1e-9	0,06		corr(6,8)	p < 1e-10	0,10
7_13_Hz_5	p < 1e-10	0,20		corr(1,4)	p < 1e-7	0,05		corr(6,9)	p < 1e-10	0,13
13_25_Hz_5	p < 1e-10	0,22		corr(1,5)	p < 1e-10	0,07		corr(7,8)	p < 1e-10	0,08
25_35_Hz_5	p < 1e-10	0,21		corr(1,6)	p < 1e-10	0,07		corr(7,9)	p < 1e-10	0,10
35_45_Hz_5	p < 1e-10	0,21		corr(1,7)	p < 1e-6	0,05		corr(8,9)	p < 1e-10	0,10
