# Supplementary material for: Novel Candidate Genes Associated with Hippocampal Oscillations
Source: PLoS One. 2011 Oct 31;6(10):e26586. doi: 10.1371/journal.pone.0026586 (PMC3204991; doi:10.1371/journal.pone.0026586)
Supplement: Table S2 — Heritability scores ( h ) and P- values from F statistics from the ANOVAs of all the traits derived in the carbachol condition (oscillations). The trait names are coded: Amplitude a_b_Hz_c indicates the integrated amplitude between a and b Hz, in region c. Amplitude_a is the peak amplitude in region a, Frequency_a indicates the peak frequency in region a. PLF(a,b) is the phase locking factor of the activity between region a and region b. The numbers refer to the following regions: 1 = CA3 stratum radiatum/lacunosum moleculare, 2 = CA3 stratum pyramidale, 3 = CA3 stratum oriens, 4 = CA1 stratum radiatum/lacunosum moleculare, 5 = CA1 stratum pyramidale, 6 = CA1 stratum oriens, 7 = Dentate Gyrus hilus, 8 = Dentate Gyrus stratum granulosum, 9 = Dentate Gyrus stratum moleculare. (XLS) [file pone.0026586.s021.xls]

trait	ANOVA	*h*		trait	ANOVA	*h*		trait	ANOVA	*h*
1_4_Hz_1	p < 1e-7	0,06		25_35_Hz_5	p < 1e-10	0,12		PLF(1,2)	p < 1e-3	0,03
4_7_Hz_1	p < 1e-8	0,07		35_45_Hz_5	p < 1e-10	0,16		PLF(1,3)	p < 0.13	0,01
7_13_Hz_1	p < 1e-9	0,07		Amplitude _5	p < 1e-10	0,10		PLF(1,4)	p < 1e-10	0,09
13_25_Hz_1	p < 1e-7	0,06		Frequency_5	p < 1e-10	0,22		PLF(1,5)	p < 1e-7	0,06
25_35_Hz_1	p < 1e-8	0,06		1_4_Hz_6	p < 0.16	0,01		PLF(1,6)	p < 1e-5	0,04
35_45_Hz_1	p < 1e-10	0,13		4_7_Hz_6	p < 1e-7	0,06		PLF(1,7)	p < 1e-9	0,07
Amplitude _1	p < 1e-10	0,08		7_13_Hz_6	p < 1e-10	0,09		PLF(1,8)	p < 1e-8	0,07
Frequency_1	p < 1e-10	0,23		13_25_Hz_6	p < 1e-10	0,11		PLF(1,9)	p < 1e-5	0,05
1_4_Hz_2	p < 1e-6	0,06		25_35_Hz_6	p < 1e-10	0,11		PLF(2,3)	p < 0.37	0,00
4_7_Hz_2	p < 1e-7	0,06		35_45_Hz_6	p < 1e-10	0,14		PLF(2,4)	p < 1e-9	0,07
7_13_Hz_2	p < 1e-7	0,06		Amplitude _6	p < 1e-10	0,07		PLF(2,5)	p < 1e-7	0,06
13_25_Hz_2	p < 1e-7	0,06		Frequency_6	p < 1e-10	0,19		PLF(2,6)	p < 1e-5	0,05
25_35_Hz_2	p < 1e-10	0,08		1_4_Hz_7	p < 1e-3	0,02		PLF(2,7)	p < 1e-7	0,06
35_45_Hz_2	p < 1e-10	0,17		4_7_Hz_7	p < 1e-5	0,05		PLF(2,8)	p < 1e-6	0,05
Amplitude _2	p < 1e-10	0,09		7_13_Hz_7	p < 1e-5	0,05		PLF(2,9)	p < 1e-6	0,05
Frequency_2	p < 1e-10	0,22		13_25_Hz_7	p < 1e-6	0,05		PLF(3,4)	p < 1e-5	0,04
1_4_Hz_3	p < 1e-4	0,04		25_35_Hz_7	p < 1e-8	0,07		PLF(3,5)	p < 1e-5	0,05
4_7_Hz_3	p < 1e-6	0,05		35_45_Hz_7	p < 1e-10	0,15		PLF(3,6)	p < 1e-8	0,06
7_13_Hz_3	p < 1e-7	0,06		Amplitude _7	p < 1e-10	0,08		PLF(3,7)	p < 0.01	0,02
13_25_Hz_3	p < 1e-9	0,07		Frequency_7	p < 1e-10	0,20		PLF(3,8)	p < 0.01	0,02
25_35_Hz_3	p < 1e-10	0,10		1_4_Hz_8	p < 0.05	0,02		PLF(3,9)	p < 0.04	0,02
35_45_Hz_3	p < 1e-10	0,16		4_7_Hz_8	p < 1e-4	0,04		PLF(4,5)	p < 1e-6	0,05
Amplitude _3	p < 1e-10	0,10		7_13_Hz_8	p < 1e-5	0,05		PLF(4,6)	p < 1e-4	0,04
Frequency_3	p < 1e-10	0,22		13_25_Hz_8	p < 1e-7	0,06		PLF(4,7)	p < 1e-10	0,08
1_4_Hz_4	p < 1e-5	0,04		25_35_Hz_8	p < 1e-8	0,07		PLF(4,8)	p < 1e-9	0,08
4_7_Hz_4	p < 1e-8	0,07		35_45_Hz_8	p < 1e-10	0,12		PLF(4,9)	p < 1e-6	0,05
7_13_Hz_4	p < 1e-8	0,06		Amplitude _8	p < 1e-8	0,07		PLF(5,6)	p < 1e-5	0,05
13_25_Hz_4	p < 1e-7	0,06		Frequency_8	p < 1e-10	0,22		PLF(5,7)	p < 1e-7	0,06
25_35_Hz_4	p < 1e-10	0,08		1_4_Hz_9	p < 1e-4	0,04		PLF(5,8)	p < 1e-8	0,07
35_45_Hz_4	p < 1e-10	0,17		4_7_Hz_9	p < 1e-5	0,04		PLF(5,9)	p < 1e-5	0,05
Amplitude _4	p < 1e-10	0,10		7_13_Hz_9	p < 1e-4	0,04		PLF(6,7)	p < 1e-6	0,05
Frequency_4	p < 1e-10	0,21		13_25_Hz_9	p < 1e-6	0,05		PLF(6,8)	p < 1e-9	0,07
1_4_Hz_5	p < 1e-3	0,03		25_35_Hz_9	p < 1e-8	0,06		PLF(6,9)	p < 1e-5	0,04
4_7_Hz_5	p < 1e-10	0,08		35_45_Hz_9	p < 1e-10	0,08		PLF(7,8)	p < 1e-4	0,04
7_13_Hz_5	p < 1e-10	0,09		Amplitude _9	p < 1e-10	0,09		PLF(7,9)	p < 0.01	0,02
13_25_Hz_5	p < 1e-10	0,09		Frequency_9	p < 1e-10	0,21		PLF(8,9)	p < 1e-3	0,03
