# Supplementary material for: Novel Candidate Genes Associated with Hippocampal Oscillations
Source: PLoS One. 2011 Oct 31;6(10):e26586. doi: 10.1371/journal.pone.0026586 (PMC3204991; doi:10.1371/journal.pone.0026586)
Supplement: Table S3 — Description and IDs of first subset of phenotypes from the GeneNetwork phenotype database: physiological hippocampal traits. (XLS) [file pone.0026586.s022.xls]

**Description of the phenotype**	**GeneNetwork ID**
Hippocampus mossy fiber pathway volume, total [units]	10362
Central nervous system, morphology: Hippocampus mossy fiber pathway volume, CA4 (dentate gyrus) component [volume units]	10363
Hippocampus mossy fiber pathway volume, suprapyramidal component (SPMF) [units]	10364
Hippocampus mossy fiber pathway volume, intra- and infrapyramidal component (IIPMF) [units]	10365
Central nervous system, morphology: Hippocampus mossy fiber pathway, ratio of volume of CA4 to total MF [%]	10366
Hippocampus mossy fiber pathway volume, fraction of suprapyramidal component relative to total volume [%]	10367
Hippocampus mossy fiber pathway volume, fraction of IIPMT relative to total volume [%]	10368
Central nervous system, morphology: Hippocampus weight, bilateral, fixed and hand-dissected tissue [mg]	10375
Central nervous system, morphology: Hippocampus bilateral weight [mg]	10376
Central nervous system, morphology: Hippocampus granule cell density [n x10^3]	10377
Central nervous system, morphology: Hippocampus granule cell number, unilateral [n x10^3]	10378
Central nervous system, morphology: Hippocampus volume, total [mm^3]	10456
Central nervous system, morphology: Hippocampus proper volume [mm^3]	10457
Central nervous system, morphology: Hippocampus pyramidal cell layer volume, unilateral [mm^3]	10458
Central nervous system, morphology: Hippocampus dentate gyrus granule cell layer volume, unilateral [mm^3]	10459
Central nervous system, morphology: Hippocampus dentate gyrus volume [mm^3]	10460
High affinity choline uptake (concentration of 0.5*10^-6M) in hippocampus [pmole/4min/mg protein]	10607
Central nervous system, morphology, aging: Polyglucosan bodies in the hippocampus of 18-month-old females [density]	10685
Dorsal hippocampus volume residuals, age adjusted [mm^3]	10755
Hippocampus, ventral hippocampus volume, age adjusted residuals (V2 Table 2A) [mm^3]	10756
Ventral hippocampus volume residuals, adjusted for differences in age and brain weight (from V3 Table 2A) [mm^3]	10757
Hippocampus volume, bilateral, in situ postmortem from high field MRI [mm^3]	10895
Iron level in hippocampus of males [ug/g]	11027
Iron level in hippocampus of females [ug/g]	11028
Copper level in hippocampus of males [ug/g wet tissue]	11029
Copper level in hippocampus of females [ug/g wet weight]	11030
Zinc level in hippocampus of males [ug/g]	11031
Zinc level in hippocampus of females [ug/g]	11032
Nrxn1 expression in hippocampus, principal component trait 1, January 2010; exon probe sets only from hippocampus Exon ST	12557
Hippocampus mossy fiber pathway volume, total without IMF (hilus + SMF) [mm^3]	12587
Hippocampus mossy fiber pathway volume, infrapyramidal (IMF) [mm^3]	12588
Hippocampus mossy fiber pathway volume, suprapyramidal (SMF) [mm^3]	12589
Hippocampus mossy fiber pathway volume, hilus [mm^3]	12590
Hippocampus mossy fiber pathway volume, total (hilus + IMF + SMF) [mm^3]	12591
