# Supplementary material for: Novel Candidate Genes Associated with Hippocampal Oscillations
Source: PLoS One. 2011 Oct 31;6(10):e26586. doi: 10.1371/journal.pone.0026586 (PMC3204991; doi:10.1371/journal.pone.0026586)
Supplement: Table S4 — Description and IDs of second subset of phenotypes from the GeneNetwork phenotype database: behavioral traits. (XLS) [file pone.0026586.s023.xls]

Description of the phenotype	GeneNetwork ID
Fear conditioning response, activity during third tone-shock pairing for males [units]	11399
Novel open field behavior, locomotion in the center from 0-15 min for males [cm]	11510
Novel open field behavior, locomotion in the center from 0-15 min for males [n beam breaks]	11506
Novel open field behavior, locomotion from 5-10 min [cm]	10916
Total distance travelled day 1 of novel environment [cm]	10037
Anxiety assay, untreated baseline, percentage of entries into closed arms of a plus maze for males [%]	11456
Anxiety assay, baseline untreated control (BASE group), activity in closed quadrants using an elevated zero maze in 60 to 120-day-old males only during last 5 min [beam breaks/sec]	12353
Locomotion, distance traveled in 30 min in open field, baseline [cm]	10416
Novel open field behavior, locomotion from 0-30 min [cm]	10910
Anxiety assay, baseline untreated control (BASE group), activity in closed quadrants using an elevated zero maze in 60 to 120-day-old males only during 10 min [beam breaks/sec]	12354
Novel open field behavior, locomotion in the center from 0-60 min for males [n beam breaks]	11500
Fear conditioning response, activity during third tone-shock pairing for males and females [units]	11913
Novel open field behavior, locomotion in the center from 0-60 min for males [cm]	11501
Novel open field behavior, locomotion from 10-15 min [cm]	10912
Novel open field behavior, locomotion from 20-25 min [cm]	10914
Fear conditioning behavior, baseline activity in apparatus for males [units]	11393
Novel open field behavior, locomotion in the center from 15-30 min for males [n beam breaks]	11507
Fear conditioning response, activity during second tone-shock pairing for males and females [units]	11912
Locomotion in both compartments of a light-dark box for males and females [cm]	11900
Novel open field behavior, locomotion in the center from 15-30 min for males [cm]	11511
Fear conditioning response, activity during second tone-shock pairing for males [units]	11398
Novel open field behavior, locomotion in the center from 0-15 min for males and females [cm]	12024
Locomotion in both compartments of a light-dark box for males [cm]	11386
Locomotion in dark compartment of a light-dark box for males and females [cm]	11898
Fear conditioning response, baseline activity in apparatus for males and females [units]	11907
Locomotion in dark compartment of a light-dark box for males [cm]	11384
Novel open field behavior, locomotion in the center from 0-15 min for males and females [n beam breaks]	12020
Novel open field behavior, locomotion from 15-20 min [cm]	10913
Fear conditioning response, activity after second tone-shock pairing for males [n beam breaks/30 sec test]	11401
Open field behavior, locomotion from 0-5 min for males and females [cm]	11921
Fear conditioning, baseline preconditioning freezing during a 2 min acclimation period prior to first pairing of conditioned stimulus (CS) and unconditioned stimulus (US) [%]	10899
Anxiety assay, baseline untreated control (BASE group), activity in closed quadrants using an elevated zero maze in 60 to 120-day-old males only during first 5 min [beam breaks/sec]	12352
Anxiety assay, baseline untreated control (BASE group), time in open quadrants using an elevated zero maze in 60 to 120-day-old males and females during 10 min [percentage of time]	12358
Anxiety assay, locomotion in the light compartment of a light-dark box for males and females [cm]	11901
Fear conditioning response, contextual activity for males and females [units]	11908
Anxiety assay, transitions between light and dark sides of a light-dark box for males and females [n]	11905
Anxiety assay, transitions between light and dark sides of a light-dark box for males [n]	11391
Novel open field behavior, locomotion in the center from 30-45 min for males [n beam breaks]	11508
Anxiety assay, baseline untreated control (BASE group), activity in closed quadrants using an elevated zero maze in 60 to 120-day-old males and females during last 5 min [beam breaks/sec]	12363
Anxiety assay, baseline untreated control (BASE group), time in open quadrants using an elevated zero maze in 60 to 120-day-old males and females during first 5 min [percentage of time]	12356
Fear conditioning response, contextual activity for males [units]	11394
Open field behavior, locomotion from 0-5 min for females [cm]	11664
Anxiety assay, baseline untreated control (BASE group), time in open quadrants using an elevated zero maze in 60 to 120-day-old males and females during last 5 min [percentage of time]	12357
Open field behavior, locomotion from 5-10 min for males and females [cm]	11924
Anxiety assay, time in closed arms of an elevated plus maze for males [sec]	11466
Anxiety assay, untreated baseline, percent time in closed arms of a plus maze for males [%]	11458
Open field behavior, locomotion for entire test period for males and females [cm]	11932
Fear conditioning response, activity after third tone-shock pairing for females [n beam breaks/30 sec test]	11659
Fear conditioning response, activity after first tone shock pairing for males [n beam breaks/30 sec test]	11400
Anxiety assay, locomotion in the light compartment of a light-dark box for males [cm]	11387
Fear conditioning response, activity after second tone-shock pairing for males and females [n beam breaks/30 sec test]	11915
Open field behavior, locomotion from 5-10 min for males [cm]	11410

Locomotion in zero maze for males [n beam breaks]	11433
Fear conditioning response, activity during first tone-shock pairing for males [units]	11397
Novel open field behavior, locomotion in the center from 0-60 min for males and females [n beam breaks]	12014
Locomotion in both compartments of a light-dark box for females [cm]	11643
Novel open field behavior, locomotion in the center from 30-45 min for males [cm]	11512
Open field behavior, locomotion from 10-15 min for males [cm]	11408
Anxiety assay, baseline untreated control (BASE group), time in open quadrants using an elevated zero maze in 60 to 120-day-old females only during last 5 min [percentage of time]	12337
Open field behavior, locomotion from 0-5 min for males [cm]	11407
Novel open field behavior, locomotion in the center from 0-60 min for males and females [cm]	12015
Open field behavior, locomotion for entire test period for males [cm]	11418
Anxiety assay, locomotion in the light compartment of a light-dark box for females [cm]	11644
Open field behavior, locomotion from 10-15 min for males and females [cm]	11922
Novel open field behavior, locomotion from 25-30 min [cm]	10915
Fear conditioning response, activity after first tone-shock pairing for males and females [n beam breaks/30 sec test]	11914
Anxiety assay, baseline untreated control (BASE group), activity in closed quadrants using an elevated zero maze in 60 to 120-day-old males only during last 5 min [n beam breaks]	12350
Novel open field behavior, locomotion from 0-5 min [cm]	10911
Anxiety assay, baseline untreated control (BASE group), time in open quadrants using an elevated zero maze in 60 to 120-day-old females only during 10 min [percentage of time]	12338
Locomotion in zero maze for males and females [n beam breaks]	11947
Novel open field behavior, locomotion in the center from 45-60 min for males [n beam breaks]	11509
Learning and memory performance, difference in latency to reach the platform between days 1 and 4 of acquisition of task using Morris water maze [s]	10816
Fear conditioning response, activity after third tone-shock pairing for males and females [n beam breaks/30 sec test]	11916
Fear conditioning response, activity during first tone-shock pairing for males and females [units]	11911
Locomotion in dark compartment of a light-dark box for females [cm]	11641
Learning and memory performance, difference in swim path to reach the platform between days 1 and 4 of acquisition of task using Morris water maze [cm]	10815
Novel open field behavior, locomotion in the center from 0-15 min for females [cm]	11767
Anxiety assay, baseline untreated control (BASE group), activity in closed quadrants using an elevated zero maze in 60 to 120-day-old males and females during 10 min [n beam breaks]	12361
Open field behavior, locomotion from 15-20 min for males and females [cm]	11923
Novel open field behavior, locomotion in the center from 0-15 min for females [n beam breaks]	11763
Anxiety assay, baseline untreated control (BASE group), time in open quadrants using an elevated zero maze in 60 to 120-day-old males only during 10 min [percentage of time]	12348
Anxiety assay, baseline untreated control (BASE group), activity in closed quadrants using an elevated zero maze in 60 to 120-day-old males and females during last 5 min [n beam breaks]	12360
Novel open field behavior, locomotion in the center from 15-30 min for males and females [n beam breaks]	12021
Novel open field behavior, locomotion in the center from 45-60 min for males [cm]	11513
Anxiety assay, baseline untreated control (BASE group), activity in closed quadrants using an elevated zero maze in 60 to 120-day-old males and females during 10 min [beam breaks/sec]	12364
Anxiety assay, baseline untreated control (BASE group), time in open quadrants using an elevated zero maze in 60 to 120-day-old males only during first 5 min [percentage of time]	12346
Anxiety assay, baseline untreated control (BASE group), activity in closed quadrants using an elevated zero maze in 60 to 120-day-old males only during 10 min [n beam breaks]	12351
Anxiety assay, baseline untreated control (BASE group), activity in closed quadrants using an elevated zero maze in 60 to 120-day-old males and females during first 5 min [n beam breaks]	12359
Anxiety assay, baseline untreated control (BASE group), latency to enter an open quadrant using an elevated zero maze in 60 to 120-day-old males and females [sec]	12355
Open field behavior, locomotion for entire test period for females [cm]	11675
Anxiety assay, transitions between light and dark sides of a light-dark box for females [n]	11648
Open field behavior, locomotion from 15-20 min for males [cm]	11409
Anxiety assay, untreated baseline, percentage of entries into closed arms of a plus maze for males and females [%]	11970
Novel open field behavior, locomotion in the center from 15-30 min for males and females [cm]	12025
Fear conditioning response, suppression of activity in altered context for males and females [units]	11910
Novel open field behavior, locomotion in the center from 0-60 min for males and females [n beam breaks]	11867
Anxiety assay, baseline untreated control (BASE group), time in open quadrants using an elevated zero maze in 60 to 120-day-old females only during first 5 min [percentage of time]	12336
Open field behavior locomotion from 5-10 min for females [cm]	11667
Anxiety assay, baseline untreated control (BASE group), activity in closed quadrants using an elevated zero maze in 60 to 120-day-old females only during first 5 min [n beam breaks]	12339
Novel open field behavior, locomotion in the center from 45-60 min for males and females [n beam breaks]	12023

Anxiety assay, baseline untreated control (BASE group), time in open quadrants using an elevated zero maze in 60 to 120-day-old males only during last 5 min [percentage of time]	12347
Fear conditioning response, activity during third tone-shock pairing for females [units]	11656
Novel open field behavior, locomotion in the center from 0-60 min for males and females [cm]	11868
Fear conditioning response, suppression of activity in altered context for males [units]	11396
Anxiety assay, time in light side of a light-dark box for females [sec]	11645
Anxiety assay, time in dark side of a light-dark box for females [sec]	11642
Novel open field behavior, vertical activity (rears) from 15-30 min in the center for males [n beam breaks]	11515
Novel open field behavior, vertical activity (rears) from 0-60 min in the center for males [n beam breaks]	11502
Novel open field behavior, locomotion in the center from 30-45 min for males and females [n beam breaks]	12022
Fear conditioning response, baseline activity in apparatus for females [units]	11650
Novel open field behavior, locomotion in the center from 0-60 min for females [n beam breaks]	11610
Open field behavior, percentage of distance in the perimeter for males and females [%]	11933
Open field behavior, percentage of distance in the center for males and females [%]	11918
Anxiety assay, percentage of time in the light side of a light-dark box for females [%]	11647
Novel open field behavior, locomotion in the periphery from 0-60 min for males [n beam breaks]	11531
Fear conditioning response, activity during second tone-shock pairing for females [units]	11655
Novel open field behavior, vertical activity (rears) from 0-15 min in the center for males [n beam breaks]	11514
Novel open field behavior, locomotion in the center from 45-60 min for males and females [cm]	12027
Fear conditioning response, activity in altered context during presentation of cue for males [n beam breaks]	11395
Novel open field behavior, vertical activity (rears) in the periphery from 45-60 min for males [n beam breaks]	11529
Novel open field behavior, locomotion in the periphery from 0-60 min for males [cm]	11532
Novel open field behavior, locomotion in the center from 0-60 min for males [cm]	11354
Novel open field behavior, percentage of locomotion in the periphery for males [%]	11530
Novel open field behavior, vertical activity (rears) from 45-60 min in the center for males [n beam breaks]	11517
Fear conditioning, freezing response to conditioned cue after 24 hours (%)	11010
Open field behavior, percentage of distance in the perimeter for males [%]	11419
Open field behavior, percentage of distance in the center for males [%]	11404
Novel open field behavior, locomotion in the center from 0-60 min for males [n beam breaks]	11353
Anxiety assay, baseline untreated control (BASE group), activity in closed quadrants using an elevated zero maze in 60 to 120-day-old females only during 10 min [n beam breaks]	12341
Novel open field behavior, locomotion in the center from 30-45 min for males and females [cm]	12026
Fear response, response to contextual fear [% freezing]	10446
Novel open field behavior, locomotion in the center from 0-60 min for females [cm]	11611
Novel open fled behavior, habituation measured as the difference in locomotion between 0-5 min and 26-30 min periods for males and females [cm]	10285
Life span, longevity [days] (RWW winsorized outliers BXD2, BXD14, and DBA2J from 400, 500, and 175 days, respectively)	10112
Novel open field behavior, vertical activity (rears) from 30-45 min in the center for males [n beam breaks]	11516
Fear conditioning response, activity after second tone-shock pairing for females [n beam breaks/30 sec test]	11658
Anxiety assay, arm entries (total) in an elevated plus maze [n]	10909
Novel open field behavior, vertical activity (rears) in the periphery from 30-45 min for males [n beam breaks]	11528
Anxiety assay, untreated baseline, percentage of entries into closed arms of a plus maze for females [%]	11713
Anxiety assay, time in middle of an elevated plus maze for males [sec]	11467
Anxiety assay, untreated baseline, percent entries into open arms of an elevated plus maze for males [%]	11457
Motor coordination, time on dowel over a 2 min baseline test period for females [sec]	11562
Fear conditioning response, activity in altered context during presentation of cue for males and females [units]	11909
Pain response, thermal nociception, latency to paw withdrawal and licking response using a hot plate test [sec]	10897
Anxiety assay, baseline untreated control (BASE group), latency to enter an open quadrant using an elevated zero maze in 60 to 120-day-old females only [sec]	12335
Fear response, preconditioned stimulus [% freezing]	10447
Novel open field behavior, vertical activity (rears) in the periphery from 0-60 min for males [n beam breaks]	11533
Anxiety assay, baseline untreated control (BASE group), activity in closed quadrants using an elevated zero maze in 60 to 120-day-old males only during first 5 min [n beam breaks]	12349
Open field behavior, percentage of time in center for males and females [%]	11919
Open field behavior, percentage of time in perimeter for males and females [%]	11934
Open field behavior, vertical activity (rears) from 0-5 min for females [n beam breaks]	11671
Anxiety assay, entries into open quadrants of a zero maze for males and females [n]	11949
Open field behavior, vertical activity (rears) 10-15 min for males [n beam breaks]	11415
Anxiety assay, entries in closed quadrants of a zero maze for males and females [n]	11948

Novel open field behavior, locomotion in the center from 0-60 min for females [n beam breaks]	11757
Anxiety assay, untreated baseline, entries into open arms of a plus maze for males [n]	11455
Anxiety assay, untreated baseline, percent time in closed arms of a plus maze for females [%]	11715
Anxiety assay, time in closed arms of an elevated plus maze for females [sec]	11723
Fear conditioning response, activity in altered context for males and females [units]	11906
Open field behavior, locomotion from 10-15 min for females [cm]	11665
Anxiety assay, untreated baseline, entries into closed arms of a plus maze for females [n]	11711
Fear conditioning response, activity in altered context for males [units]	11392
Novel open field behavior, percentage of locomotion in the periphery for males and females [%]	12044
Anxiety assay, percentage of time in light compartment of a light-dark test [%]	10907
Anxiety assay, percentage of locomotion in light side of a light-dark box for females [%]	11646
Startle response to loud acoustic stimulus	11007
Novel open field behavior, locomotion in the center from 0-60 min for females [cm]	11758
Open field behavior, vertical activity (rears) from 5-10 min for males and females [n beam breaks]	11931
Novel open field behavior, vertical activity (rears) in the periphery from 15-30 min for males [n beam breaks]	11527
Novel open field behavior, vertical activity (rears) from 45-60 min in the center for males and females [n beam breaks]	12031
Anxiety assay, open field behavior, duration in center of field [sec]	11015
Pain response, thermal nociception, 54 degree C hot plate latency baseline and post swim in maximum possible effect for males [%MPE=(postswim-baseline)/(cut-off-baseline)*100)]	10426
Anxiety assay, closed arm entries in an elevated plus maze test [n]	10896
Anxiety assay, time in the center of an elevated plus maze [sec]	10905
Open field behavior, locomotion from 15-20 min for females [cm]	11666
Fear conditioning response, activity after first tone-shock pairing for females [n beam breaks/30 sec test]	11657
Fear conditioning response, cue conditioning, activity suppression after third tone-shock pairing for females [units]	11660
Anxiety assay, open arm entries in an elevated plus maze test [n]	10898
Fear conditioning response, cue conditioning, activity suppression after third tone-shock pairing for males [units]	11403
Fear conditioning response, suppression of activity in altered context for females [units]	11653
Mean distance travelled (day 1 total distance-day 3 total distance) [cm]	10038
Open field behavior, percentage distance in the perimeter for females [%]	11676
Open field behavior, percentage of distance in the center for females [%]	11661
Open field behavior, vertical activity (rears) from 5-10 min for males [n beam breaks]	11417
Novel open fled behavior, habituation measured as the difference in locomotion between 0-5 min and 26-30 min periods for females [cm]	10332
Fear conditioning, freezing during conditioned stimulus exposure, 24 hr after conditioning [%]	10902
Open field behavior, percentage of time in perimeter for males [%]	11420
Open field behavior, percentage of time in center for males [%]	11405
Anxiety assay, time in open arms of a plus maze for males [sec]	11468
Anxiety assay, untreated baseline, percentage of time in open arms of a plus maze for males [%]	11459
Anxiety assay, baseline untreated control (BASE group), activity in closed quadrants using an elevated zero maze in 60 to 120-day-old females only during last 5 min [beam breaks/sec]	12343
Fear conditioning response, contextual activity for females [units]	11651
Anxiety assay, baseline untreated control (BASE group), activity in closed quadrants using an elevated zero maze in 60 to 120-day-old females only during last 5 min [n beam breaks]	12340
Pain response, thermal nociception, 54 degree C hot plate latency baseline and post swim in maximum possible effect for males and females [%MPE=(postswim-baseline)/(cut-off-baseline)*100)]	10424
Anxiety assay, entries in open quadrants of a zero maze for males [n]	11435
Anxiety assay, untreated baseline, percentage of time in open arms of a plus maze for females [%]	11716
Anxiety assay, entries in closed quadrants of a zero maze for males [n]	11434
Novel open field behavior, locomotion in the center from 45-60 min for females [n beam breaks]	11766
Novel open field behavior, vertical activity (rears) from 0-60 min in the center for males and females [n beam breaks]	12016
Anxiety assay, baseline untreated control (BASE group), activity in closed quadrants using an elevated zero maze in 60 to 120-day-old males and females during first 5 min [beam breaks/sec]	12362
Anxiety assay, percentage of time in open arms of an elevated plus maze [%]	10908
Open field behavior, vertical activity (rears) from 5-10 min for females [n beam breaks]	11674
Activity level, locomotion in zero maze for females [n beam breaks]	11690
Fear conditioning response, activity after third tone-shock pairing for males [n beam breaks/30 sec test]	11402
Anxiety assay, untreated baseline, percent entries into open arms of an elevated plus maze for females [%]	11714
Novel open field behavior, locomotion in the periphery from 0-60 min for males and females [n beam breaks]	12045

Anxiety assay, time in middle of an elevated plus maze for females [sec]	11724
Locomotor performance, duration on an accelerating rotarod, mean of three trials [s]	10824
Novel open field behavior, locomotion in the periphery from 0-60 min for males and females [cm]	12046
Open field behavior, vertical activity (rears) from 10-15 min for males and females [n beam breaks]	11929
Novel open field behavior, locomotion in the center from 45-60 min for females [cm]	11770
Anxiety assay, baseline untreated control (BASE group), latency to enter an open quadrant using an elevated zero maze in 60 to 120-day-old males only [sec]	12345
Motor coordination, rotarod performance, untreated training trial for males [sec]	11465
Novel open field behavior, vertical activity (rears) from 30-45 min in the center for males and females [n beam breaks]	12030
Open field behavior, percentage of time in center for females [%]	11662
Open field behavior, percentage of time in perimeter for females [%]	11677
Pain response, thermal nociception, 54 degree C hot plate latency baseline and post swim in maximum possible effect for females [%MPE=(postswim-baseline)/(cut-off-baseline)*100)]	10425
Open field behavior, time in corners for females [min]	11663
Novel open field behavior, vertical activity (rears) from 15-30 min in the center for males and females [n beam breaks]	12029
Fear conditioning, freezing response to conditioned cue after 48 hours (%)	11009
Pain response, 54 degree C hot plate latencies post-swim in male [sec]	10423
Anxiety assay, percentage of time in light side of a light-dark box for males [%]	11390
Anxiety assay, time in closed arms of an elevated plus maze for males and females [sec]	11980
Anxiety assay, untreated baseline, percent time in closed arms of a plus maze for males and females [%]	11972
Pain response, thermal nociception, 54 degree C hot plate latencies post-swim, male and female average [sec]	10421
Anxiety assay, untreated baseline, entries into closed arms of a plus maze for males and females [n]	11968
Novel open field behavior, vertical activity (rears) from 0-15 min in the center for males and females [n beam breaks]	12028
Floor preference baseline, intrinsic preference for the grid-textured floor for the control group of males [s/min]	10101
Novel open fled behavior, habituation measured as the difference in locomotion between 0-5 min and 26-30 min periods for males [cm]	10331
Anxiety assay, time in dark side of a light-dark box for males [sec]	11385
Anxiety assay, time in light side of a light-dark box for males [sec]	11388
Novel open field behavior, locomotion in the center from 30-45 min for females [n beam breaks]	11765
Pain response, 54 degree C hot plate latencies post-swim in female [seconds]	10422
Open field behavior, vertical activity (rears) from 0-20 min for males and females [n beam breaks]	11927
Floor preference baseline, intrinsic preference for the grid-textured floor for the control group of males [s/min]	10103
Open field behavior, vertical activity (rears) from 0-20 min for males [n beam breaks]	11413
Open field behavior, percentage time in perimeter minus time in corners for males [%]	11412
Novel open field behavior, locomotion in the center from 15-30 min for females [n beam breaks]	11764
Novel open field behavior, locomotion in the center from 30-45 min for females [cm]	11769
Open field behavior, vertical activity (rears) from 0-20 min for females [n beam breaks]	11670
Open field behavior, percentage of time in perimeter minus time in corners for males and females [%]	11926
Anxiety assay, entries into open quadrants of a zero maze for females [n]	11692
Anxiety assay, entries in closed quadrants of a zero maze for females [n]	11691
Novel open field behavior, vertical activity (rears) in the periphery from 0-15 min for males [n beam breaks]	11526
Anxiety assay, time in open arm of elevated plus maze [sec]	11012
Fear conditioning response, activity in altered context during presentation of cue for females [units]	11652
Open field behavior, vertical activity (rears) from 0-5 min for males and females [n beam breaks]	11928
Motor coordination, rotarod performance, untreated training trial for females [sec]	11722
Novel open field behavior, vertical activity (rears) in the periphery from 45-60 min for males and females [n beam breaks]	12043
Floor preference baseline, time on grid textured floor for experimental group of males [sec/min]	10093
Novel open field behavior, time in the field center [sec]	10906
Motor coordination, time on dowel over a 2 min baseline test period for males and females [sec]	11819
Novel open field behavior, vertical activity (rears) in the periphery from 30-45 min for males and females [n beam breaks]	12042
Open field behavior, time in corners for males and females [min]	11920
Hippocampus granule cell number [n total per side]	10337
Novel open field behavior, locomotion in the periphery from 0-60 min for females [n beam breaks]	11788
Novel open field behavior, vertical activity (rears) in the periphery from 0-15 min for females [n beam breaks]	11783
Learning and memory, spatial navigation, latency to reach platform using Morris water maze (test 1) [log	10413

sec]	
Anxiety assay, percentage of locomotion in light side of a light-dark box for males [%]	11389
Anxiety assay, baseline untreated control (BASE group), activity in closed quadrants using an elevated zero maze in 60 to 120-day-old females only during 10 min [beam breaks/sec]	12344
Novel open field behavior, locomotion in the center from 15-30 min for females [cm]	11768
Novel open field behavior, percentage of locomotion in the periphery for females [%]	11787
Novel open field behavior, vertical activity (rears) in the periphery from 0-60 min for males and females [n beam breaks]	12047
Novel open field behavior, urinations for females [n/test period]	11620
Pain response, 54 degree C hot plate latencies baseline (before 3 min forced swim in 15 degree C water), male [seconds]	10420
Novel open field behavior, locomotion in the periphery from 0-60 min for females [cm]	11789
Novel open field behavior, vertical activity (rears) in the periphery from 15-30 min for females [n beam breaks]	11784
Fear conditioning, freezing in response to context exposure 48 hr after conditioning [%]	10901
Novel open field behavior, urinations for males and females [n/test period]	11877
Novel open field behavior, vertical activity (rears) from 45-60 min for males [n beam breaks]	11352
Novel open field behavior, vertical activity (rears) from 45-60 min for males and females [n beam breaks]	11866
Central nervous system, behavior: Learning and memory function, probe trial water maze time spent in swim path, day 5 [%]	10345
Anxiety assay, untreated baseline, entries into open arms of a plus maze for females [n]	11712
Anxiety assay, time in open arms of an elevated plus maze for females [sec]	11725
Pain response, thermal nociception, 54 degree C hot plate latency baseline test (before 3 min forced swim in 15 degree C water) for females [sec]	10419
Novel open field behavior, vertical activity (rears) in the periphery from 15-30 min for males and females [n beam breaks]	12041
Novel open field behavior, vertical activity (rears) in the periphery from 45-60 min for females [n beam breaks]	11786
Novel open field behavior, vertical activity (rears) from15-30 min in the center for females [n beam breaks]	11772
Prepulse inhibition of the acoustic startle response [%]	11008
Anxiety assay, locomotion in the light compartment relative to total in a light-dark test [%]	10904
Anxiety assay, time in closed quadrants of a zero maze for males and females [sec]	11952
Anxiety assay, time in open quadrants of a zero maze for males and females [sec]	11953
Novel open field behavior, vertical activity (rears) from 45-60 min in the center for females [n beam breaks]	11774
Open field behavior, vertical activity (rears) from 10-15 min for females [n beam breaks]	11672
Anxiety assay, time in middle of an elevated plus maze for males and females [sec]	11981
Novel open field behavior, vertical activity (rears) in the periphery from 0-60 min for females [n beam breaks]	11790
Anxiety assay, time in open arms of an elevated plus maze for males and females [sec]	11982
Anxiety assay, time in open quadrants of a zero maze for males [sec]	11439
Anxiety assay, time in closed quadrants of a zero maze for males [sec]	11438
Anxiety assay, untreated baseline, entries into open arms of a plus maze for males and females [n]	11969
Anxiety assay, percentage time in open quadrants of a zero maze for males and females [%]	11951
Novel open field behavior, vertical activity (rears) from 45-60 min for females [n beam breaks]	11609
Morris water maze behavior, mean swim speed during acquisition of task [cm/s]	10814
Response to auditory stimulus US in contextualized fear conditioning paradigm [% freezing]	10445
Novel open field behavior, vertical activity (rears) from 0-15 for females [n beam breaks]	11606
Learning and memory performance, time spent in target quadrant on day 5 (probe trial) using the Morris water maze [%]	10344
Novel open field behavior, vertical activity (rears) in the center from 0-60 min for males [n beam breaks]	11355
Fear conditioning response, cue conditioning, activity suppression after third tone-shock pairing for males and females [units]	11917
Novel open field behavior, vertical activity (rears) in the center from 0-60 min for males and females [n beam breaks]	11869
Anxiety assay, percentage time in light side of a light-dark box for males and females [%]	11904
Novel open field behavior, vertical activity (rears) from 30-45 min for males [n beam breaks]	11351
Anxiety assay, percentage of time in open quadrants of a zero maze for males [%]	11437
Novel open field behavior, vertical activity (rears) from min 30-45 for males and females [n beam breaks]	11865
Fear conditioning response, activity during first tone-shock pairing for females [units]	11654
Anxiety assay, untreated baseline, percent entries into open arms of an elevated plus maze for males and females [%]	11971
Open field behavior, vertical activity (rears) from 15-20 min for males [n beam breaks]	11416
Fear conditioning, freezing response to context after 48 hours (%)	11011
Novel open field behavior, vertical activity (rears) from 15-30 min for males [n beam breaks]	11350

Learning and memory, spatial navigation, latency to reach platform using Morris water maze (test 3) [log sec]	10415
Novel open field behavior,vertical activity (rears) from 0-15 min for males and females [n beam breaks]	11863
Novel open field behavior, vertical activity (rears) in the center from 0-60 min for females [n beam breaks]	11612
Novel open field behavior, urinations for males [n/test period]	11363
Anxiety assay, time in light side of a light-dark box for males and females [sec]	11902
Anxiety assay, time in dark side of a light-dark box for males and females [sec]	11899
Motor performance, improvement in rotarod training measured as change in time on rod [sec]	11005
Open field behavior, time in corners for males [min]	11406
Motor coordination, rotarod performance, untreated training trial for males and females [sec]	11979
Fear conditioning response, activity in altered context for females [units]	11649
Anxiety assay, untreated baseline, percentage of time in open arms of plus maze for males and females [%]	11973
Anxiety assay, time in open quadrants of a zero maze for females [sec]	11696
Anxiety assay, time in closed quadrants of a zero maze for females [sec]	11695
Novel open field behavior, vertical activity (rears) from 0-15 min for males [n beam breaks]	11349
Novel open field behavior, vertical activity (rears) from 15-30 min for males and females [n beam breaks]	11864
Novel open field behavior, vertical activity (rears) in the periphery from 30-45 min for females [n beam breaks]	11785
Anxiety assay, number of closed arm entries using elevated plus maze [n]	11013
Open field behavior, locomotion [cm]	11014
% swim path spent in target quadrant	10807
Novel open field behavior, vertical activity (rears) in the periphery from 0-15 min for males and females [n beam breaks]	12040
Fear conditioning, freezing during consolidation period after final pairing of conditioned stimulus (CS) and unconditioned stimulus (US) [%]	10900
Anxiety assay, percentage of locomotion in light side of a light-dark box for males and females [%]	11903
Novel open field behavior, vertical activity (rears) from 30-45 min for females [n beam breaks]	11608
Open field behavior, vertical activity (rears) from 0-5 min for males [n beam breaks]	11414
Anxiety assay, percentage time in open quadrants of a zero maze for females [%]	11694
Novel open field behavior, vertical activity (rears) from 0-15 min in the center for females [n beam breaks]	11771
Mean latency to reach the platform [s] during acquisition of watermaze task	10808
Anxiety assay, transitions between light and dark compartments in light/dark test [n]	10917
Rotarod performance, baseline control (supplementary data to Brigman et al.) [sec]	11004
Open field behavior, vertical activity (rears) from 15-20 min for males and females [n beam breaks]	11930
Depression assay, immobility from 2-10 min in a 10 min Porsolt forced swim test [%]	10903
Motor coordination, time on dowel over a 2 min baseline test period for males [sec]	11305
Sleep need, slow wave sleep delta EEG power after 6 hrs sleep deprivation [%]	10143
Open field behavior, percentage time in perimeter minus time in corners for females [%]	11669
Learning and memory, spatial navigation, latency to reach platform using Morris water maze (test 2) [log sec]	10414
Novel open field behavior, vertical activity (rears) from 15-30 min for females [n beam breaks]	11607
Open field behavior, vertical activity (rears) from 15-20 min for females [n beam breaks]	11673
Anxiety assay, baseline untreated control (BASE group), activity in closed quadrants using an elevated zero maze in 60 to 120-day-old females only during first 5 min [beam breaks/sec]	12342
Novel open field behavior, vertical activity (rears) from 0-60 min in the center for females [n beam breaks]	11759
Anxiety assay, untreated baseline, entries into closed arms of a plus maze for males [n]	11454
Pain response, thermal nociception, 54 degree C hot plate latency baseline test (before 3 min forced swim in 15 degree C water) for males and females [sec]	10418
Novel open field behavior, vertical activity (rears) from 30-45 min in the center for females [n beam breaks]	11773
Learning and memory performance, mean path length to reach the platform during acquisition of task using Morris water maze [cm]	10810
